# Mechanistic Modeling of the Interplay Between Host Immune System, IL-7 and UCART19 Allogeneic CAR-T Cells in Adult B-cell Acute Lymphoblastic Leukemia

**DOI:** 10.1158/2767-9764.CRC-22-0176

**Published:** 2022-11-30

**Authors:** Thibaud Derippe, Sylvain Fouliard, Ibtissam Marchiq, Sandra Dupouy, Maria Almena-Carrasco, Julia Geronimi, Xavier Declèves, Marylore Chenel, Donald E. Mager

**Affiliations:** 1 Institut de Recherches Internationales Servier, Suresnes, France.; 2 Department of Pharmaceutical Sciences, University at Buffalo, State University of New York, Buffalo, New York.; 3 Université de Paris, Inserm, UMRS-1144, Optimisation Thérapeutique en Neuropsychopharmacologie, Paris, France.

## Abstract

Chimeric antigen receptor (CAR)-T cell therapies have shown tremendous results against various hematologic cancers. Prior to cell infusion, a host preconditioning regimen is required to achieve lymphodepletion and improve CAR-T cell pharmacokinetic exposure, leading to greater chances of therapeutic success. To better understand and quantify the impact of the preconditioning regimen, we built a population-based mechanistic pharmacokinetic-pharmacodynamic model describing the complex interplay between lymphodepletion, host immune system, homeostatic cytokines, and pharmacokinetics of UCART19, an allogeneic product developed against CD19^+^ B cells. Data were collected from a phase I clinical trial in adult relapsed/refractory B-cell acute lymphoblastic leukemia and revealed three different UCART19 temporal patterns: (i) expansion and persistence, (ii) transient expansion with subsequent rapid decline, and (iii) absence of observed expansion. On the basis of translational assumptions, the final model was able to capture this variability through the incorporation of IL-7 kinetics, which are thought to be increased owing to lymphodepletion, and through an elimination of UCART19 by host T cells, which is specific to the allogeneic context. Simulations from the final model recapitulated UCART19 expansion rates in the clinical trial, confirmed the need for alemtuzumab to observe UCART19 expansion (along with fludarabine cyclophosphamide), quantified the importance of allogeneic elimination, and suggested a high impact of multipotent memory T-cell subpopulations on UCART19 expansion and persistence. In addition to supporting the role of host cytokines and lymphocytes in CAR-T cell therapy, such a model could help optimizing the preconditioning regimens in future clinical trials.

**Significance:**

A mathematical mechanistic pharmacokinetic/pharmacodynamic model supports and captures quantitatively the beneficial impact of lymphodepleting patients before the infusion of an allogeneic CAR-T cell product. Mediation through IL-7 increase and host T lymphocytes decrease is underlined, and the model can be further used to optimize CAR-T cell therapies lymphodepletion regimen.

## Introduction

B-cell acute lymphoblastic leukemia (B-ALL) is a malignant proliferation of lymphoid progenitor cells that can invade bone marrow (BM), blood, and extramedullary sites. Current standard treatment consists of essentially multiagent cytotoxic therapies followed, in high-risk groups, by hematopoietic stem cell transplantation (HSCT). Although these therapies are relatively efficient in children (representing 80% of all B-ALL), with a 5-year survival rate (SR) equal to 90%, their benefits decrease substantially in middle age patients (SR = 30–55%) and those over 60 years of age (SR below 30%).

Chimeric antigen receptor (CAR)-T cell therapy is one among several innovative approaches being explored to improve B-ALL treatment (1). The goal is to increase the cytotoxicity of the host immune system to efficiently fight cancer cells, through the arming of T cells with a CAR. This synthetic receptor is a fusion between an antibody-derived single-chain variable fragment directed against a specific tumor antigen, the CD3z chain of the T-cell receptor, and one or two costimulatory molecules such as CD28 or 4-1BB (2).

The production of CAR-T cells consists of extracting human T lymphocytes with leukapheresis prior to a viral transduction of the CAR and *in vitro* expansion of the engineered cells. T cells can be derived either from the patient (autologous CAR-T cells) or a healthy donor (allogeneic CAR-T cells). All current CAR-T products on the market are autologous in nature (3–6), which confers the advantage of avoiding donor and/or recipient HLA compatibility issues. However, owing to previous treatments, T lymphocyte quality and quantity can lead to manufacturing failures and patient exclusion. Furthermore, even when T-cell production is successful, the duration of the bed-to-bed process might be too long (7, 8). Allogeneic CAR-T cells theoretically solve these problems by allowing an “off-the-shelf” access (9) based on nondamaged T cells (10). However, allogeneic CAR-T cell therapy still must overcome two major hurdles: GvHD, with CAR-T cells attacking patient cells, and host-versus-graft reaction, which refers to the rejection of the infused product by patient immune systems. Both reactions could limit CAR-T cell engraftment and long-term therapeutic efficacy.

A lymphodepleting regimen, generally consisting of fludarabine and cyclophosphamide (FC) given prior to autologous CAR-T cell infusion, is a crucial step for efficient therapy (11). Extensive evidence supports the importance of lymphodepletion in supporting CAR-T cell engraftment and expansion through different mechanisms, including the increased availability of homeostatic cytokines such as IL-7 and IL-15 (12–15) and the reduction of anti-CAR immune rejection (16).

Following lymphodepletion, there is a significant relationship between autologous CAR-T cell exposure in blood and clinical response (4, 11, 17), making it critical to identify the determinants of CAR-T cell pharmacokinetic properties. In the autologous setting, four main pharmacokinetic phases after CAR-T cell infusion have been described (18). First, injected CAR-T cells are removed from the blood through distribution to various organs (19, 20), reaching homing and egress equilibrium with second lymphoid organs (SLO) or BM. Second, CAR-T cells rapidly proliferate during an expansion phase, owing to CAR-T cell activation following specific target binding. Once the maximum peak expansion (*C*_max_) in the blood is reached, CAR-T cells decline during a rapid contraction phase followed by a more stable and persistent phase.

Several CAR-T pharmacokinetic models have been published. Adapting a model of activated nonmodified T lymphocytes, Stein and colleagues developed an empirical model structure (21), which was further enriched by Liu and colleagues (22). After expansion occurs, a fraction of effector cells is rapidly removed, whereas another fraction is transformed into memory cells with a longer half-life. Other models have a more mechanistic basis. For example, Hardiansyah and colleagues included explicit tissue distribution and a link between expansion and targeted B cells (23). The relationship between CAR affinity, antigen abundance, tumor cell depletion, and CAR-T cell expansion were also investigated by Singh and colleagues (24). Owens and colleagues explored with their theoretical model the impact of preconditioning in the safety and efficacy of autologous CAR-T cell therapies (25). However, to our knowledge, no prior published models address the relationship between lymphodepletion and allogeneic CAR-T cell pharmacokinetics.

T lymphocytes can be divided into memory cell subpopulations, such as stem cell memory (*T_SCM_*), central memory (*T_CM_*), effector memory (*T_EM_*), and terminal effector (*T_TE_*) cells (26). Each subpopulation presents with specific attributes such as lymphoid homing, stemness, and proliferation potential. Several models have been proposed regarding the differentiation from one subpopulation to another (27). In the linear model, effector cells are expanding with some of them secondarily evolving into long-lasting memory cells. In contrast, the progressive differentiation model describes T cells evolving from *T_SCM_* to effector cells (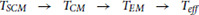) (28, 29). *T_SCM_* cells are under particular investigation for their self-renewal and multipotent capacities to reconstitute the entire spectrum of memory and effector subsets property (26, 30) and for their positive impact on CAR-T cell expansion (31).

**FIGURE 1:**
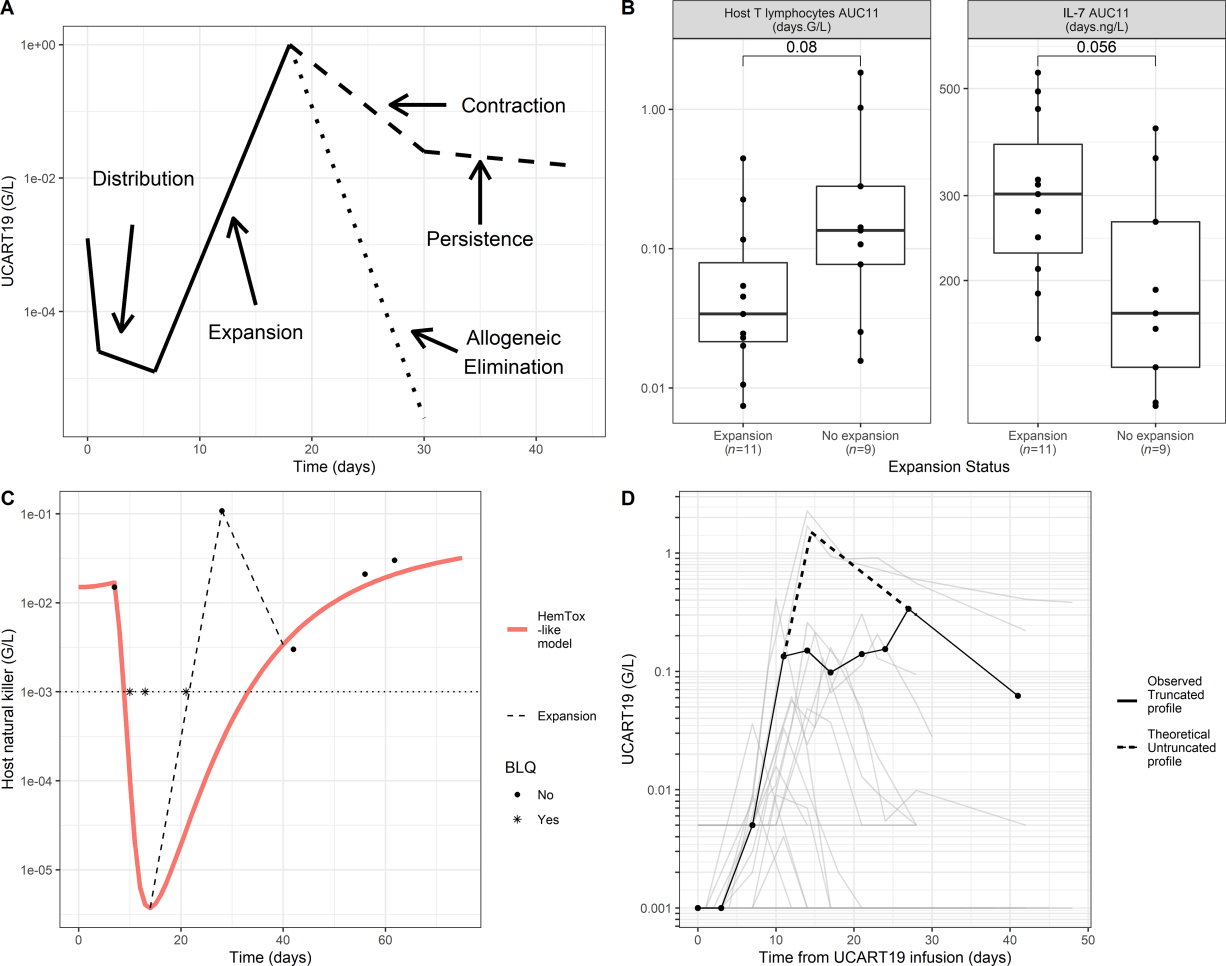
Main features of the model. **A,** CAR-T cell phases: UCART19 first distributes from blood to tissue before expansion. Afterward, some sets undergo a fast allogeneic elimination, while others experience contraction and persistence. **B,** IL-7 and host T early AUC (from day 0 to day 11 after UCART19 infusion, day 11 being the median of UCART19 Tmax—measured by flow cytometry) versus expansion status, using the Wilcoxon test. High IL-7 and low host T exposures are related to higher expansions. **C,** One individual host NK-cell profile illustrating the theoretical decomposition of host lymphocytes (NK and host T cells work the same way): red curve represents a HemTox_like model, able to capture both initial and final observations. However, it does not capture an additional peak of lymphocyte observed around UCART19’s Tmax. An additional expansion of lymphocytes was thus added in the model (dotted curve). Host T/NK are thus the sum of these two systems. **D,** Illustrative example of a UCART19 truncated profile in blood.

UCART19 is an allogeneic CAR-T cell product (32) developed against relapsed/refractory (R/R) CD19^+^ B-cell ALL. This therapy was tested during CALM and PALL phase I clinical trials (6, 33). Briefly, three patterns of UCART19 pharmacokinetic profiles were observed: (i) an expansion followed by long persistence, (ii) an expansion followed by a sharp decline after the *C_max_*, and (iii) an absence of observed expansion (Fig. 1, A). The importance of lymphodepletion was verified as none of the three patients receiving only a FC protocol had CAR-T cell expansion or clinical response. The addition of anti-52 mAb alemtuzumab (FCA) was further required to observe UCART19 cell expansion, which was associated with early blood exposures of both IL-7 and host T cells (Fig. 1B). UCART19 is theoretically not targeted by alemtuzumab because of its TALEN-mediated CD52 KO. Additionnal features were added in order to capture elaborate patterns such as transient peaks (Fig. 1C) or truncated profiles (Fig. 1D).

The primary objective of this study is to develop a mechanistic model to characterize the complex interplay between UCART19, host lymphocytes, IL-7 concentrations, and lymphodepleting agents. A secondary objective was to explore the dynamics of UCART19 memory cell subpopulations by using a progressive differentiation model.

## Materials and Methods

This section first provides key information regarding the clinical trial protocol and bioanalytical assays previously described in several publications (6, 33). The model construction is then described in detail, including the generation of the assumptions, their mathematical translations into a set of ordinary equations, and an overview of the global model building strategy.

### CALM Study

Data were collected from the CALM phase I study (Trial registration ID: NCT02746952), which involved 25 adults with R/R CD19^+^ B-cell ALL. All patients received UCART19 once, except 3 patients who received a second dose approximately 80 days after the first one. Given the small number of patients having undergone redosing and the long interval between doses, all pharmacokinetic and pharmacokinetic/pharmacodynamic information collected after each dosing event was considered a distinct set of data, resulting in 28 pharmacokinetic/pharmacodynamic sets. Two sets were discarded after exploratory analysis because of an analytic error and an outlier lymphocyte profile (described in Supplementary Fig. S1). The remaining sets were used in model development and evaluation (*n* = 26, corresponding to 23 patients). Three dose levels of UCART19 were infused: 6 × 10^6^ cells (*n* = 6), 6–8 × 10^7^ cells (*n* = 12), and 1.8–2.4 × 10^8^ cells (*n* = 8). All sets received three doses of fludarabine [median = 165 mg (range, 120–275 mg)] between day −7 and day −5 (from UCART administration) and three doses of cyclophosphamide [median = 2,670 mg (range, 2,010–4,802 mg)] between day −4 and day −2. All sets, except two, also received alemtuzumab, either as a flat dose (40 mg, *n* = 8, or 60 mg, *n* = 5) or a body weight–adjusted dose (1 mg/kg, *n* = 11), split into five doses administered from day −7 to day −3. Further details are available in an associated publication (33). The CALM study was conducted in accordance with the Declaration of Helsinki, International Conference on Harmonization, and Good Clinical Practice Guidelines and was approved by Institutional Review Boards/Ethics Committees. Written informed consent was obtained from all patients prior to inclusion in the study.

### Bioanalysis

Alemtuzumab serum concentration was determined by ELISA. Complete pharmacokinetic profiles after the last administration were available for all 24 sets (median = 11 samples per set). Flow cytometry was performed to measure levels of UCART19 (in all 26 sets), total lymphocytes (26 sets), host natural killer (NK) cells (25 sets), and host T lymphocytes (20 sets) in blood. The limit of quantification (LOQ) for the flow cytometry assay was 0.001 G/L for most sets, but greater for a select few (depending on sample analysis locations). Alemtuzumab LOQ was 0.01 μg/mL. IL-7 plasma concentration, determined by sandwich electrochemiluminescence assay, was available in 20 sets. All observations made after a new drug administration posterior to the last UCART19 infusion (HSCT preconditioning drugs for instance) were discarded.

### Model Development

#### Exploratory Analysis

Prior to the modeling process, an exploratory analysis was performed through visualization of time profiles of variables and correlation between covariates and derived descriptive parameters. Detailed results are described in an associated publication (33).

#### Overview of the Assumptions

According to the exploratory analysis, the following assumptions were made to guide the development of the structural model:

- Lymphodepleting agents (fludarabine, cyclophosphamide, and alemtuzumab) eliminate both host NK cells and host T lymphocytes, with no effect on UCART19 (given the short half-life of F and C, and the presence of CD52 KO on UCART19 that prevents alemtuzumab effects).
- Host T lymphocytes are able to recognize and eliminate allogeneic UCART19. This allogeneic response is not systematic.
- Host T lymphocytes negatively regulate IL-7 exposure, whereas IL-7 supports UCART19 expansion.
- UCART19 cells are split into several memory cell subpopulations following a progressive differentiation model.

**FIGURE 2:**
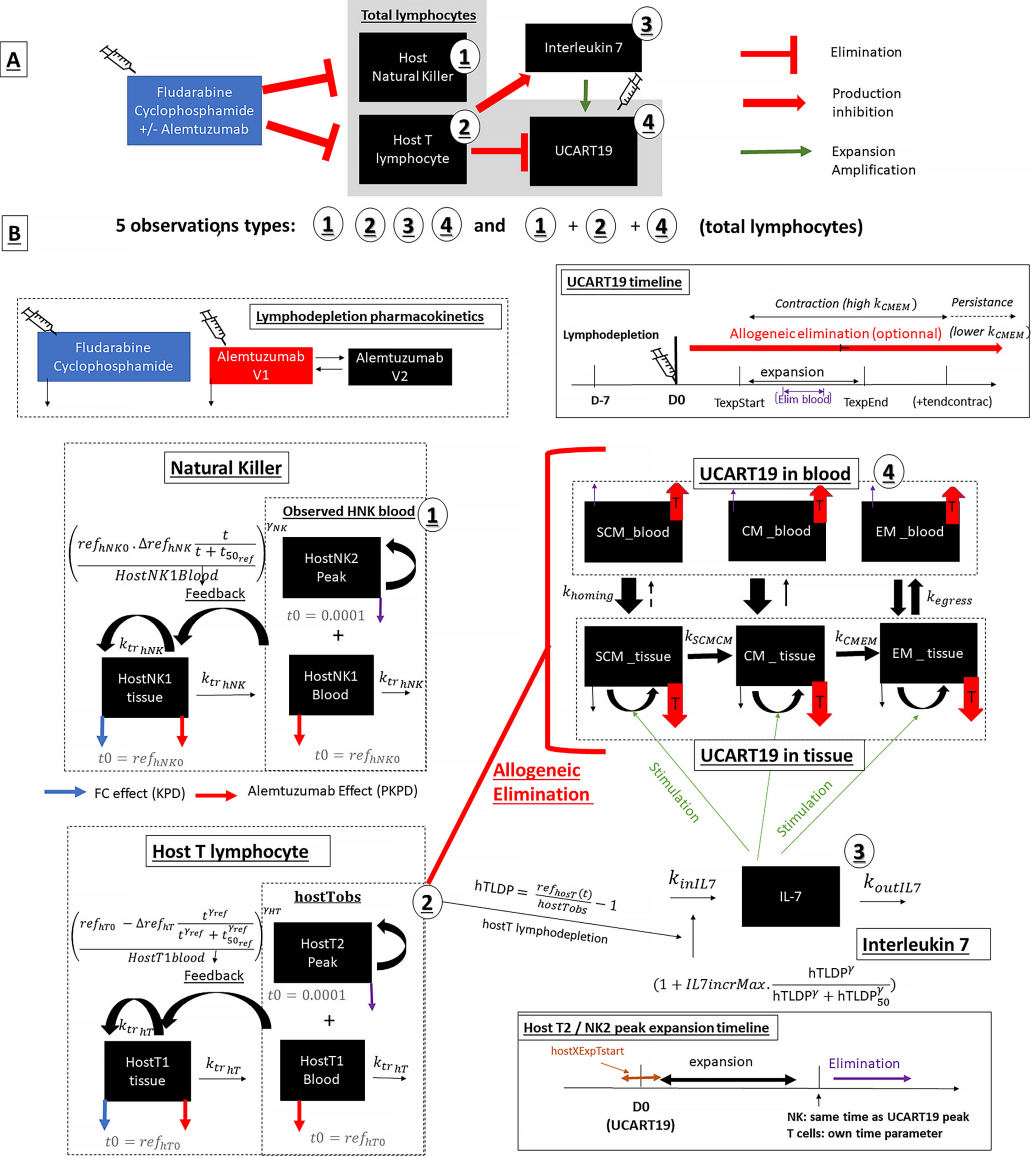
Schematics of the final mechanistic pharmacokinetic/pharmacodynamic model. **A,** General overview of the final model. Lymphodepleting agents eliminate host NK and host T cells. Host T-cell depletion allow UCART19 cells to expand both by directly decreasing host T-cell cytotoxicity and by increasing IL-7 concentrations. **B,** Detailed model schematic: Top left: Pharmacokinetics of alemtuzumab, fludarabine, and cyclophosphamide compartments. Left: host lymphocytes, divided into T and NK cells. Lymphocytes recovers with the basal system (HemTox like model), FC acts only in tissue compartments, and alemtuzumab acts both in tissue and blood compartments. An expanding lymphocyte compartment was added to capture peaks. Bottom right: timeline of expanding lymphocytes (hostX means hostT or hostNK). Above: IL-7 was modeled with a simple indirect response model with an increased production rate in case of host T lymphodepletion. Middle right: UCART19 system and each subpopulation (T_SCM_, T_CM_, and T_EM_ cells) exist in blood and tissue. Model follows a progressive differentiation model (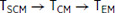). UCART19 expansion is increased by IL-7, whereas host T cells act on each UCART19 compartment. Top right: timeline of UCART19 kinetics (allogeneic elimination, expansion, and elimination in blood—for two sets only—and switch from a contraction to persistence phase).

A mathematical model based on a series of ordinary differential equations was developed according to these assumptions, describing alemtuzumab pharmacokinetics, lymphodepleting agent effects owing to host lymphocytes (NK and T cells), IL-7 increase due to lymphodepletion and UCART19 pharmacokinetics (see Fig. 2A and B for simplified and detailed schematic overviews of the final pharmacokinetic/pharmacodynamic model). The model was iteratively refined to describe the desired features of the data. Key equations are provided below, and the complete set of equations is available in the Monolix code (Supplementary Code S1).

#### Alemtuzumab and FC Pharmacokinetics

Alemtuzumab pharmacokinetic data were fitted using a two-compartment pharmacokinetic model. In accordance with the literature, both linear (34) and Michaelis–Menten (35, 36) elimination functions were investigated. Because of the lack of F and C pharmacokinetic data, FC lymphodepletion protocol was modeled using a single virtual compartment (so-called KPD model). The corresponding dose was based on normalized individual dosing information (
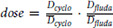
, with 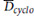 and 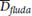 representing the median dose in CALM for each drug) and administered 7 days before UCART19 infusion.

#### Lymphocytes (host T and NK cells)

Only T cells among host lymphocytes impact UCART19 kinetics, yet host T data are missing for 6 patients. Host NK and total lymphocytes data were thus added in the model, such as host T data can be indirectly assessed (total lymphocyte - host NK – UCART19, with B cells being neglectable). Lymphodepletion was described, independently on NK and T cells, using maturation models with feedback loops [HemTox-like models (37)]:



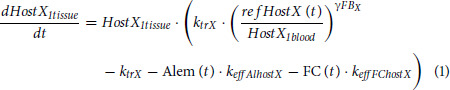





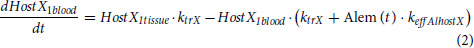



with *X* representing NK or T cells, *k_trX_* is a first-order rate constant for lymphocyte production in tissue, transfer from tissue to blood, and final elimination, and 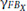 is a feedback loop parameter. Alem(*t*) represents the concentrations of alemtuzumab leading to lymphocyte elimination (mediated by *k_effAlhostX_*) both in tissue and blood compartments. FC(*t*) represents the fludarabine and cyclophosphamide virtual concentrations leading to lymphocyte elimination (mediated by *k_effFChostX_*) in the tissue compartment. Lymphocyte counts after recovery were substantially different from counts before lymphodepletion, and a time-dependent reference value was used in the feedback equation: 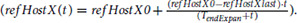 The term *refHostXlast* was estimated, whereas *refHostX* was inputted as the initial observation of NK or T cells.

HemTox-like models (*hostX*_1_) were able to describe the initial and final features of the profiles but failed to capture transient peaks (Fig. 1C). Thus, an additional compartment per T/NK (*hostX*_2_) was introduced:



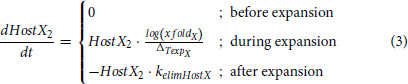



with *xfold_X_* as the magnitude of the host T2/NK2 expansions, *k_elimHostX_* is a first-order elimination rate constant and 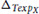 is the duration of the expansion. Total host T/NK cells are then the sum of expanding and slowly recovering HemTox cells in blood (*hostXblood = HostX*_2_ + *HostX_1blood_*).

#### IL-7 Plasma Concentration Profiles

IL-7 plasma concentrations were modeled through an indirect response model, with a stimulation of the production by host T lymphodepletion (38), expressed as the ratio 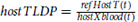:



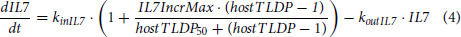



with *k_inIL7_* as a zero-order production rate constant, *k_outIL7_* is a first-order elimination rate constant, *IL7IncrMax* the maximal fold increase in IL-7 production, and *hostTLDP*_50_ is the level of *hostTLDP* – 1) producing half of *IL7IncrMax*. Host T cells were preferred over total lymphocytes for controlling IL-7 production as exploratory analysis revealed a better correlation and is in agreement with the literature (39–41).

#### Overview of the Kinetics of UCART19

Circulating UCART19 is described as the sum of three memory subpopulations in blood (i.e., *T_SCM_* + *T_CM_* + *T_EM_* cells, effectors were not integrated into the model), although no measurement of specific memory subpopulations was used. The dose of UCART19 is not a driver of the magnitude of expansion (6). To avoid a nonrealistic pharmacokinetic dose-proportionality in the model, the dose was set to a nominal value of 6.10^6^ cells for all of the sets (i.e., the lowest dose level used). Thus, the mechanistic part of the model is not designed to account for the absence of dose-expansion relationship of UCART19 (possibly caused by a maximal capacity of engraftment). The percentage of memory subpopulations was available for each infusion of UCART19 and allowed simultaneous infusions of *T_SCM_*, *T_CM_*, and *T_EM_* cells in respective blood compartments.

As defined in the model, after UCART19 administration, cells undergo rapid distribution into tissues. Cells then expand, manifesting as a strong and rapid proliferation during a short period of time. This expansion increases with IL-7 concentration. UCART19 cells also undergo an allogeneic elimination, mediated by host T lymphocytes (in blood and SLO). Therefore, sets with the smallest magnitude of lymphodepletion never reach UCART19 counts above LOQ, resulting in flat below the limit of quantification (BLQ) profiles. However, sets with greater lymphodepletion show an observable but transient expansion. Finally, five sets do not present with allogeneic elimination, explaining their persistent profiles (Fig. 1A). For these sets, a decrease in the *T_CM_* to *T_EM_* differentiation creates the transition between contraction and persistence phases (EM elimination was set to be greater than that for *T_CM_* cells). Elimination occurring only in blood was added and allowed for “truncated profiles” (Fig. 1D).

#### UCART19 Distribution and Physiologic Elimination

After UCART19 infusion, the transfers between blood and tissue (such as in SLO or BM) are mediated through *k_egressX_* and *k_homingX_* parameters, with *X* representing each memory subpopulation. Each subpopulation undergoes a physiologic elimination in tissue through the *k_elimX_* parameter. Cells follow a progressive differentiation model, with *T_SCM_* turning to *T_CM_* cells (mediated through *k_SCMCM_* parameter) and *T_CM_* turning to *T_EM_* cells. Differentiation of *T_CM_* into *T_EM_* is greater during UCART19 expansion and several days afterward. This leads to a fast first elimination rate of UCART19 (contraction phase), owing to a greater natural elimination of *T_EM_* cells. This high *T_CM_* to *T_EM_* transformation then returns to a basal level to create the persistence phase:



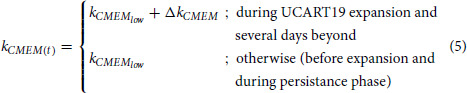



#### UCART19 Expansion Mechanism

Expansion was modeled with a first-order growth rate occurring between two timepoints. In accordance to Cieri and colleagues (28), *T_SCM_* cells have greater proliferation abilities than *T_CM_* and *T_EM_* cells. In addition, IL-7 increases the magnitude of this expansion. The expansion rate of *T_SCM_* cells in tissue (SCMexp) was defined as:



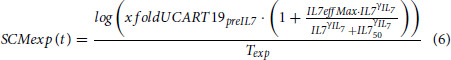



with *T_exp_* being the total duration of the expansion, *xfoldUCART19_preIL7_* is the magnitude of UCART19 proliferation in the absence of IL-7, *IL7effMax* is the maximal multiplying factor of *xfoldUCART19_preIL7_*, *IL7_50_* is the IL-7 concentration provoking half of *IL7effMax*, and 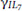 is the corresponding Hill coefficient. Given the magnitude of this expansion, a safeguard system was necessary (see method in Supplementary Materials and Methods) to avoid supraphysiologic high UCART19 counts.

Expansion rates of *T_CM_* and *T_EM_* cells (CMexp and EMexp) were derived from SCMexp:



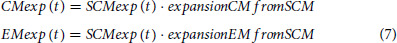



with both *expansion CM from SCM* and *expansion EM from SCM* inferior to 1.

#### UCART19 Elimination Pathways

An elimination mediated by host T lymphocytes was applied to UCART19 (both in blood and tissue) for all but five sets (for which allogeneic elimination was set to 0). For two of the five sets without allogeneic elimination, a third type elimination was added only in blood and for a short period of time, to account for the above-mentioned truncated profiles.

#### UCART19 Overall Equations

The dynamics of the UCART19 *T_CM_* subpopulation were defined as:



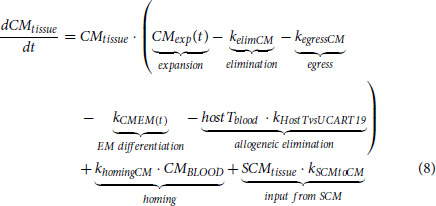





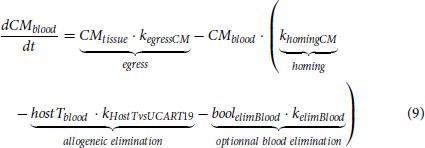



*T_SCM_* and *T_EM_* cells were assumed to behave similarly.

#### Model-building Strategy

A sequential approach by individual population parameter was used to first search for alemtuzumab individual pharmacokinetic parameters, and these values were incorporated into the main model. A population-based approach (i.e., nonlinear mixed effects modeling) was then used, which focused on characterizing average profiles (typical values) and the variability across individuals. The model-building strategy aimed at incorporating mechanistic features despite the few number of available profiles. As a result, the model combined parameters freely estimated in Monolix (version 2019R; http://www.lixoft.com), and select parameters were fixed to solve identifiability issues (a sensitivity analysis was performed with the final model and parameters). Data that were BLQ values were treated as censored data [Beal M3 method (42)]. Qualification of the model was based on goodness of fit (GOF) plots, individual fitted profiles, and population model predictive performance (based on Monte Carlo simulations).

### Data Availability Statement

The data generated in this study are not publicly available as they include information that may compromise patient privacy. They may be available for scientific and medical professions upon reasonable request and following assessment of the request. Request should be sent to the corresponding author (sylvain.fouliard@servier.com).

## Results

**FIGURE 3:**
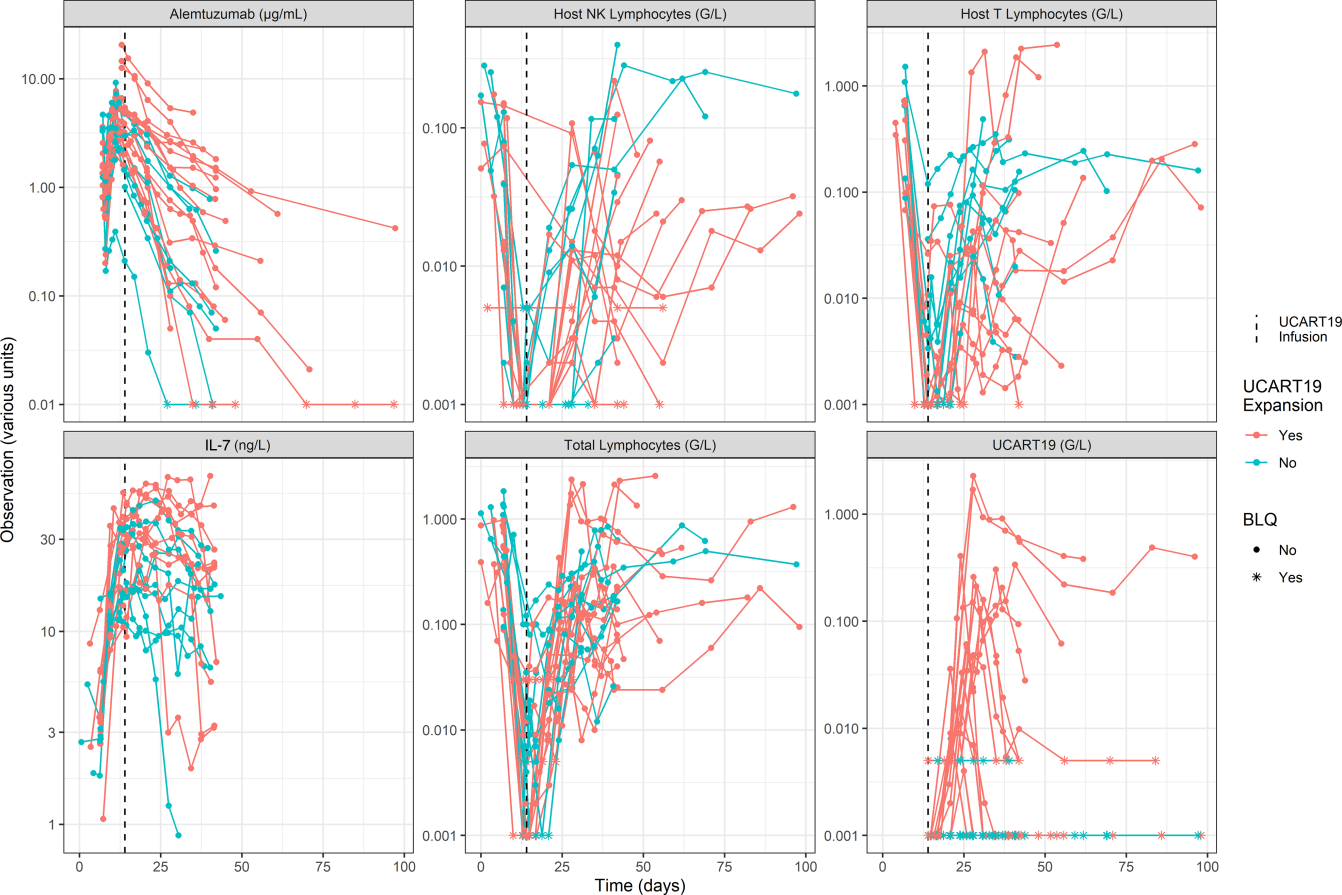
Observations used to support model building: alemtuzumab, IL-7 concentrations, total lymphocytes, host NK, host T, and UCART19 data.

**FIGURE 4:**
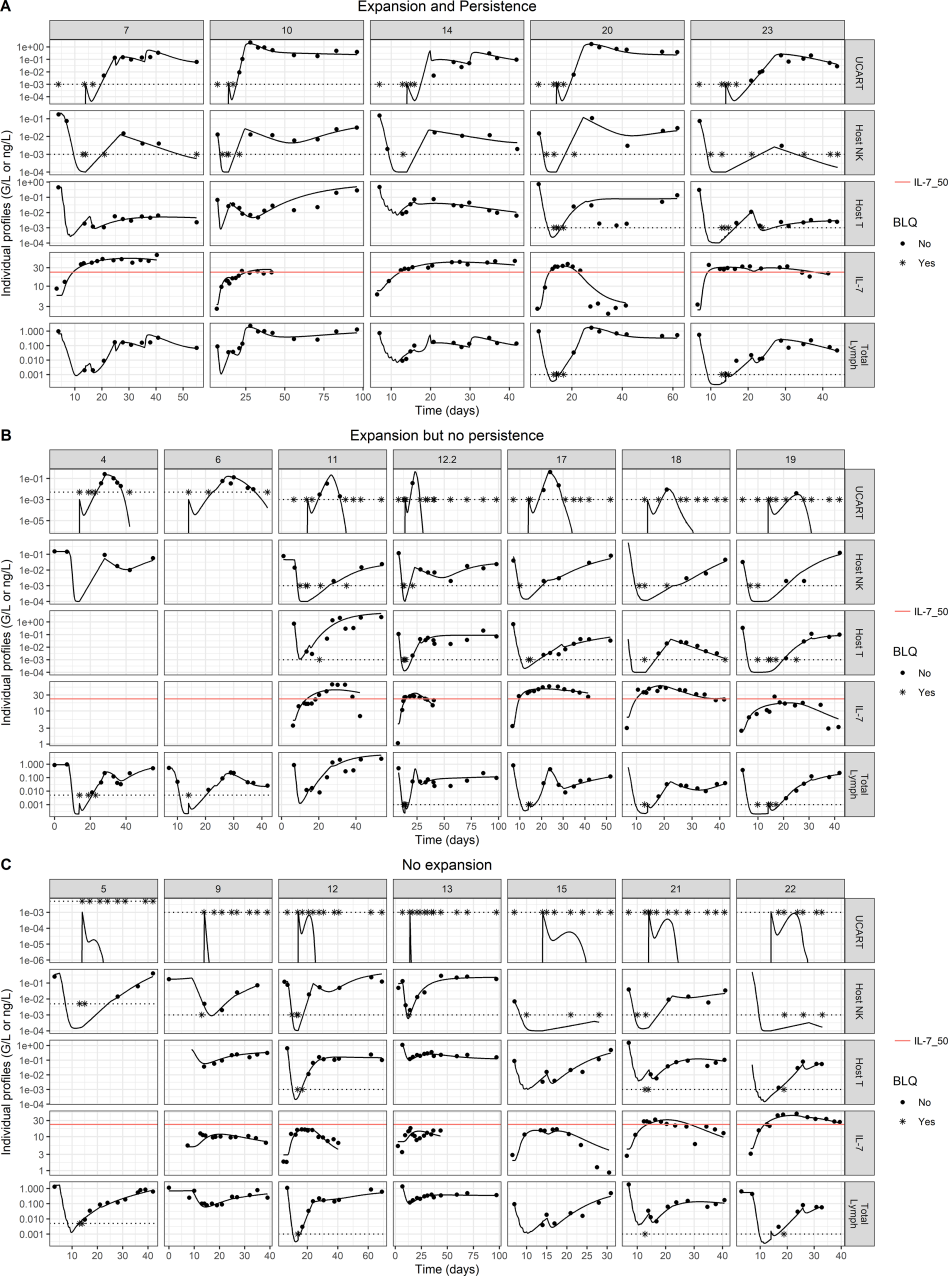
Sample of individual predictions. Each bloc represents a pattern of sets: sets with persistence (**A**), with expansion followed by fast elimination (**B**), with no expansion (**C**). Inside each block, each row represents an observation type. Dots represents observations while full line represent modeled profiles and horizontal dashed black line represent LOQ values.

All pharmacokinetic/pharmacokinetic profiles that were modeled are shown in Fig. 3. The final model included 51 population parameters, 18 of which were freely estimated. Variabilities were applied on 23 parameters. All parameter estimations with relative standard errors and parameter descriptions are presented in the Supplementary Table S1. A sample of individual predictions is presented in Fig. 4, and all profiles are available in Supplementary Fig. S2. All GOF plots are shown in Supplementary Fig. S3.

### Alemtuzumab

On the basis of the Monolix objective function and model diagnostics, the pharmacokinetics of alemtuzumab was best described with a two-compartment model and a linear systemic elimination. Estimated parameters were total clearance CL (μ = 0.96, IIV = 130%), central volume of distribution V1 (μ = 3.74, IIV = 48%), peripheral volume of distribution V2 (μ = 6.01, IIV = 71%), and intercompartmental clearance Q (μ = 3.51, IIV = 3858%). The model captured well all individual profiles (Supplementary Fig. S4A). Alemtuzumab pharmacokinetic parameters presented with high intersubject variability across all terms [in agreement with literature estimates (34, 36)], and also showed high covariance between Q, CL, and V2 parameters. An analysis of random effects suggested that alemtuzumab might exhibit target-mediated drug disposition (TMDD; ref. 43). The apparent clearance and volume of distribution (especially V2) were greater with the lowest alemtuzumab dose and with a greater tumor burden (Supplementary Fig. S4D). As empirical Bayesian estimate–derived individual profiles were judged acceptable with a sequential modeling approach and taking into account both the complexity of the main model and the lack of data to explore it further, a specific TMDD system was not tested. Model comparison with previous publications (34–36) is complex considering Alemtuzumab pharmacokinetic variability and the low number of patients in the CALM study.

### Host Lymphocytes

Samples of host NK- and T-cell profiles are shown in Fig. 4 (second and third rows). Host NK- and T-cell individual predictions were satisfactory for all but one host T-cell profile. The HemTox-like models efficiently captured initial lymphodepletion and final recovery. Second-order elimination rates of FC (2 and 0.75 days^–1^FC^–1^ for NK and on host T) were fixed without variability (given the lack of sets with the FC protocol). The effects of alemtuzumab, estimated at 0.334 on NK cells (IIV = 90%) and 1.253 on host T cells (IIV = 66%) mL · μg^–1^ · day^–1^, were precisely estimated. High interindividual variability was required to describe well all of the profiles, especially on baseline parameters (IIV around 1800% both for host NK and T cells final baselines). This is consistent with the large variability observed in the original dataset.

The HemTox-like models were not able to exhibit transient peaks during lymphodepletion until a specific compartment of expanding cells was added. Variabilities applied on the expansion range and elimination after expansion were also important, especially for the magnitude of NK-cell expansion (IIV = 1945%).

### IL-7

A sample of IL-7 profiles is shown in Fig. 4 (fourth row). Overall, the data were well described with an indirect response model. The maximal fold increase in IL-7 production, *IL7IncrMax*, was estimated to be 9.7 (IIV = 42%), with *hostTLDP50* fixed to 10 (IIV = 66%).

### UCART19

**FIGURE 5:**
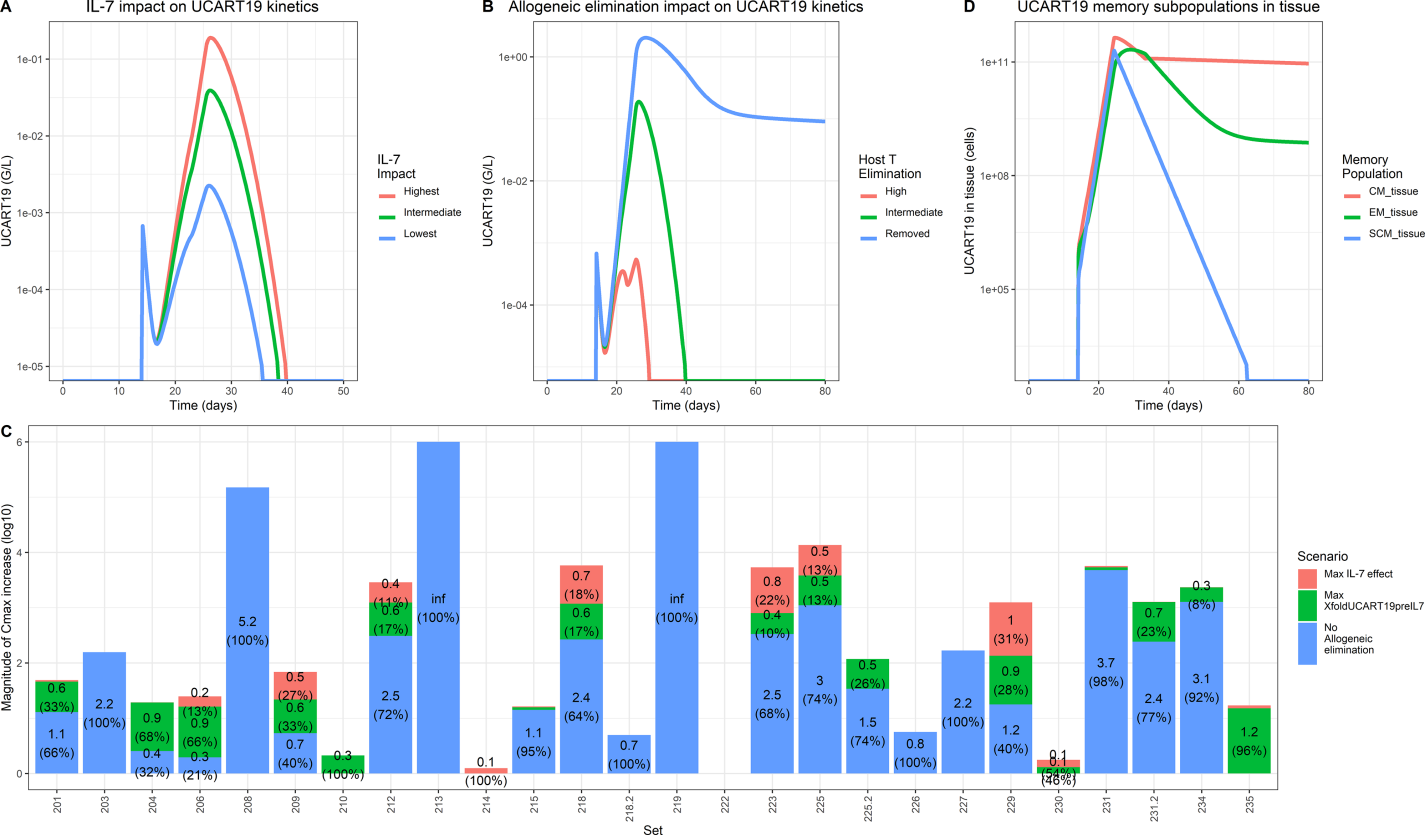
**A,** Impact of IL-7 on UCART19 profile. Simulation of a typical profile with lowest, intermediate, and highest values for IL7effMax: IL-7 increases the magnitude of the expansion. **B,** Impact of Allogeneic elimination on UCART19 profiles. In blue, the absence of allogeneic elimination leads to profiles with persistence. In green the allogeneic elimination is lower than UCART19 expansion and a transient peak is observed. In red, a high allogeneic elimination leads to the absence of observable expansion. **C,** For each set, contribution of IL-7, intrinsic proliferation capacity and allogeneic elimination on UCART19 Cmax. Y axe represent the number of *C*_max_ log_10_ increase (from individual profile) in case of maximal IL-7 effect (red), maximal intrinsic proliferation capacity (green) or absence of allogeneic elimination (blue). **D,** Memory subpopulation in tissue for a profile with persistence. In blue T_SCM_ expand faster than the other but is also transformed into T_CM_ (T_SCM_ has the highest total disappearance rate). After the expansion, T_CM_ disappears faster than T_EM_ due to a high transformation into T_EM_. This transformation is then reduced, and T_CM_ ultimately controls the persistence phase.

A sample of UCART19 profiles is shown in Fig. 4 and all three patterns of individual profiles were well described, namely (i) an expansion followed by persistence, (ii) a transient expansion, and (iii) the absence of an expansion. The impacts of IL-7 and host T lymphocytes on UCART19 pharmacokinetics were explored both on the typical profile (Fig. 5A and B, respectively) and on each individual (Fig. 5C), highlighting the overall higher impact of host T lymphocytes over IL-7 on UCART19 *C*_max_ and exposure. Figure 5D shows the memory cell subpopulation dynamics in tissue. Most of the parameters regulating this interplay were fixed on values providing good individual fits and population properties, due to identifiability issues. However, values were established such as *T_CM_* cells control the terminal slope (through a high *T_SCM_* cell differentiation rate into *T_CM_* and a greater *T_EM_* cell elimination rate). In this configuration, the role of *T_SCM_* is to provide *T_CM_* and *T_EM_* cell populations, before returning to a low value (adding a low *T_CM_* to *T_SCM_* transfer would limit *T_SCM_* cells final disappearance). The simulated impact of *T_SCM_* removal from the infusion is reported in Supplementary Fig. S5A and is dependent on the *expansionCMfromSCM* parameter. The faster *T_SCM_* cells expand as compared to *T_CM_* cells, the more UCART19 exposure is reduced in case of *T_SCM_* cell removal. Inversely, owing to the ability of *T_SCM_* cells to regenerate all other subpopulations, removing *T_CM_* and *T_EM_* cells from the product had little impact on simulated UCART19 exposure (Supplementary Fig. S5B).

**FIGURE 6:**
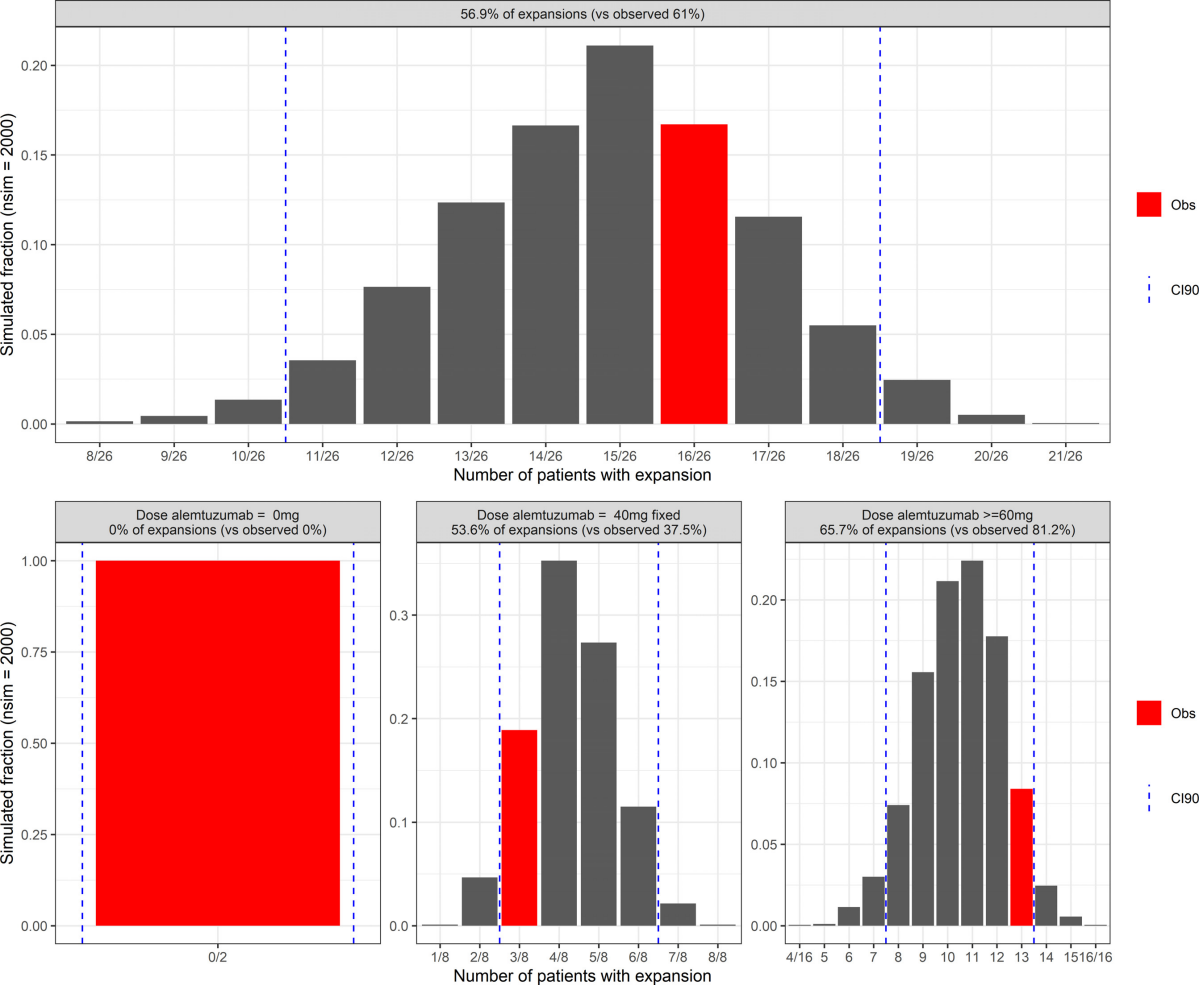
Fraction of UCART19 expansion from model simulation. For each simulation (*n* = 2,000), number of expansions was computed and reported into histograms. Observation was highlighted in red, and CI90 reported with dotted lines. Top, All sets gathered. Bottom, Sets grouped by alemtuzumab doses (0 mg, 40 mg, or higher).

The main simulation-based diagnostics of the final model are depicted in Fig. 6. Traditional visual predicted checks or associated tools were not considered useful given the variability of temporal patterns and the high volume of censored data. Instead, a numerical predictive check approach was preferred and the occurrence rate of UCART19 expansions in each simulation was computed, with the distribution compared with the observed expansion rate. Simulations led to 57% of UCART19 expansions, which agreed with the similar observed rate of 61%. When stratified by alemtuzumab dosing conditions, simulations without alemtuzumab systematically showed an absence of expansions, in accordance with observations, and simulations with a total alemtuzumab dose equal to 40 mg and equal to or greater than 60 mg resulted in 54% and 66% expansions, versus the observed values 37% and 81%. All computed values are inside the 90% confidence interval 90 (CI90).

## Discussion

### Overview

CAR-T cell therapies have generated considerable interest in treating cancer, with more success with hematologic malignancies than solid tumors. Two distinctive approaches exist regarding the source of T lymphocytes. Autologous CAR-T cells, for which the patient is also the donor, require a patient-specific and long manufacturing process. Nevertheless, they guarantee a self-recognition of the product. In contrast, allogeneic CAR-T cells allow for an “off-the-shelf” product (9), but might be rejected by the host immune system. This difference translates into different pharmacokinetic profiles, and more specifically, a shorter persistence of allogeneic CAR-T cells. Regardless of the autologous or allogeneic source of CAR-T cells, the relationship between CAR-T cell exposure and objective response is consistently observed (6, 18, 44). In this analysis, a mechanistic population-based pharmacokinetic/pharmacodynamic model was developed to provide insights into the role of lymphodepletion for allogeneic CAR -T cell expansion, focusing on the description of the interplay between lymphodepleted host and infused immune cells.

### Parameter Identifiability

The description of UCART19 cell subpopulations required the fixing of several parameters, based on values providing a good fit to the data. An alternative approach would have been to use specific parameter values from the literature (as fixed values or as priors in a Bayesian framework) under the assumption that CAR-T cells behave like normal lymphocytes. This was not the preferred approach, given the allogeneic nature of UCART19. A complete sensitivity analysis is available in Supplementary Fig. S6, which showed that the first 10 most model sensitive parameters are either related to host T cells or IL-7 concentrations. Eight of them were freely estimated. All other parameters showed little to no effect on CAR-T cell exposures, with most of their values being fixed. The correlation between IL-7 and the AUC of early host T cells was a challenge, as it confounded the identifiability and the precise segregation of the impact of each entity on UCART19 expansion. Simultaneous fits of allogeneic and autologous CAR-T cell data might help solving this issue.

### IL-7 and IL-15 Integration

Cytokines IL-7 and IL-15 are known to be involved respectively in naïve and memory lymphocytes in healthy subjects and acting together in case of lymphodepletion (45). Many studies suggest that increasing these cytokines is the real purpose of lymphodepletion prior to CAR-T therapy (12–15). IL-15 was not added into the final model because exploratory analysis showed an absence of correlation with UCART19 expansion (33), which is in contrast to prior studies (13, 46).

### IL-7 Alternative Models

Several ways to integrate IL-7 into the final model were investigated. First, IL-7 acting on the UCART19 growth rate would result in a variation in CAR-T cell profiles associated with a return of IL-7 concentrations toward baseline values, which was not observed (Supplementary Fig. S7A). Thus, IL-7 impacts the first days following CAR-T cell infusion only. An alternative way to describe the early UCART19 profiles was to introduce another elimination pathway, accounting for early rejection of engrafted UCART19 cells, occurring early after administration and being inhibited by IL-7 (Supplementary Fig. S7B). This model exhibited a similar predictive performance as the final model and is physiologically plausible.

### Allogeneic Elimination

Three reasons support the allogeneic elimination of UCART19. First, 11 of the 16 sets with expansion experienced a rapid elimination right after the end of UCART19 expansion, which is hardly observed in autologous infusions. Also, the necessity to use alemtuzumab to achieve expansion suggests a greater importance of lymphodepletion in the case of allogeneic CAR-T cells as compared with autologous infusions, and thus indicates an allogeneic elimination mechanism. Finally, three sets without expansion had IL-7 concentrations that exceeded the IL-7 threshold, suggesting that IL-7 alone was not the right driver for expansion. Adding host T cell–mediated allogeneic elimination solved those misspecifications and allowed the description of all UCART19 profiles. Thus, the model supports the role of host T lymphocytes as a UCART19 killing agent, in accordance with both literature (16) and exploratory analyses. Probably owing to the low sample size, no biomarker or covariate relationships were associated with sets that showed persistence and were not affected by this allogeneic elimination.

### Progressive Differentiation Model

In the final model, a progressive differentiation scheme explained the relationships between *T_SCM_*, *T_CM_*, and *T_EM_* cells. A high *k_SCMCM_* value leads to *T_SCM_* cells disappearing faster than *T_CM_* cells after the expansion. In this system, *T_CM_* cells control the persistence slope. An alternative configuration with a greater *T_CM_* death rate and a lower *k_SCMCM_* term would lead to *T_SCM_* cells controlling the terminal slope of every other subpopulation. Such a model was able to capture the data but was judged less physiological. In this configuration, the impact of *T_SCM_* cells removal would be even more important for UCART19 exposure. Also, effector cells were not integrated into the model because (i) no to very low effector levels were observed within UCART19 and (ii) three memory cell subpopulations allowed for the description of the variety of patterns and there was no benefit from adding effectors in the system.

### Capturing the Contraction and Persistence Phases

Sets with long persistence had their contraction and persistence phases modeled by modifying the transformation of *T_CM_* into *T_EM_* cells. The hypothesis behind this system is that during the expansion and shortly thereafter, the transformation rate of *T_CM_* into *T_EM_* cells is greater than in nonimmune reactions. In general, *T_EM_* cells have a greater death rate than *T_CM_* cells, but during the contraction phase, the *T_CM_* cell subpopulation decreases faster than *T_EM_* cells because of the differentiation of *T_CM_* into *T_EM_* cells (Fig. 5D). After this phase, the natural persistence of *T_CM_* cells drives the UCART19 profile. The impact of 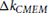 is presented in Supplementary Fig. S8. This framework is similar to that in Stein and colleagues model (21), but with a progressive differentiation framework. However, the absence of UCART19 subpopulation data (*T_SCM_*, *T_CM_*, *T_EM_*) led to uncertainty in the structural model. Indeed, alternative ways to describe the contraction phase are possible, such as changing the homing/egress ratio due to less chemoattractant (refs. 47, 48; see alternative model in Supplementary Fig. S9), or adding a new elimination pathway accounting for activation induced cell death (49) or exhaustion mechanisms (ref. 50; Supplementary Fig. S10).

### Truncated Profiles

Two sets experienced truncated profiles, captured by an additional elimination occurring only in the blood compartment. Supplementary Figure S11 highlights the impact of this elimination. This was necessary to obtain a delay between the end of UCART19 expansion and the time to reach the *C_max_*, by allowing for a decrease of circulating UCART19 with limited effect on the total system. Increasing the variability of the expansion duration was not a viable alternative as it did not allow for capturing initial observations with good fidelity. The exact nature of this elimination mechanism is unknown.

### Potential Uses of the Model

The relatively good performance of the model supports its set of assumptions, consolidating the knowledge regarding the importance of increasing IL-7 levels and reducing host lymphocytes through proper lymphodepletion to increase UCART19 expansion and exposure. In addition, the model can be used to perform simulations with alternative lymphodepletion regimens, as illustrated in Supplementary Fig. S12. For example, it is possible to test fractionations of the same alemtuzumab dose (Supplementary Fig. S12A, showing no differences between five, three, or two administrations) and several time windows of the lymphodepletion (Supplementary Fig. S12B, showing the negative impact of lymphodepleting the patients too early or too late).

## Conclusion

In summary, a translational mechanistic pharmacokinetic/pharmacodynamic model was developed to highlight some key components of the complex interplay between host immune system and UCART19, using host T cells and IL-7 concentrations as primary elements of expansion. The allogeneic CAR-T cell mechanistic pharmacokinetic/pharmacodynamic model focuses on the impact of lymphodepletion, production of homeostatic cytokines, and allogeneic elimination of UCART19, based on data collected from the CALM clinical trial. IL-7 production and allogeneic elimination by host T cells well captured all temporal patterns of UCART19 profiles, from long persistence to lack of expansion. Many other elements of CAR-T cell therapy were not taken into account, such as inflammatory cytokines (modeled in refs. 23, 24), 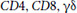 subtypes, costimulatory domain. Capturing all aspects of response to CAR-T cell treatment within the same framework is highly challenging, especially when observations vary across clinical trials. Thus, model-based analyses and data integration, with a focus on specific aspects of CAR-T therapy, represents the best way to proceed prior to further attempts to integrate all of these aspects into a unique computational framework, used to improve patient CAR-T cell therapies by optimizing their lymphodepletion regimens or finding predictive biomarkers.

## Supplementary Material

Supplementary Data S1 Supplementary Data
