# Supplementary material for: Mechanistic Modeling of the Interplay Between Host Immune System, IL-7 and UCART19 Allogeneic CAR-T Cells in Adult B-cell Acute Lymphoblastic Leukemia
Source: Cancer Res Commun. 2022 Nov 30;2(11):1532–44. doi: 10.1158/2767-9764.CRC-22-0176 (PMC10036133; doi:10.1158/2767-9764.CRC-22-0176)
Supplement: Supplementary Data S1 — Supplementary Data [file crc-22-0176-s01.docx]

Supplement Mechanistic modeling of the interplay between host immune system, interleukin 7 and UCART19 allogeneic CAR-T cells in adult B-cell acute lymphoblastic leukemia.

Authors: Thibaud Derippe ^1,2,3^, Sylvain Fouliard ^1^, Ibtissam Marchiq ^1*^, Sandra Dupouy ^1*^, Maria Almena-Carrasco ^1^, Julia Geronimi ^1^, Xavier Declèves ^3^, Marylore Chenel ^1->4**^, Donald E. Mager ^2^

^1^ Institut de Recherches Internationales Servier, Suresnes, France

^2^ Department of Pharmaceutical Sciences, University at Buffalo, State University of New York, Buffalo, New York.

^3^ Université de Paris, Inserm, UMRS-1144, Optimisation Thérapeutique en Neuropsychopharmacologie, F-75006 Paris

^4^ Pharmetheus AB, Uppsala, Sweden

* Equal contribution; ** Marylore Chenel left Servier for Pharmetheus during this project

## N patients / sets of data


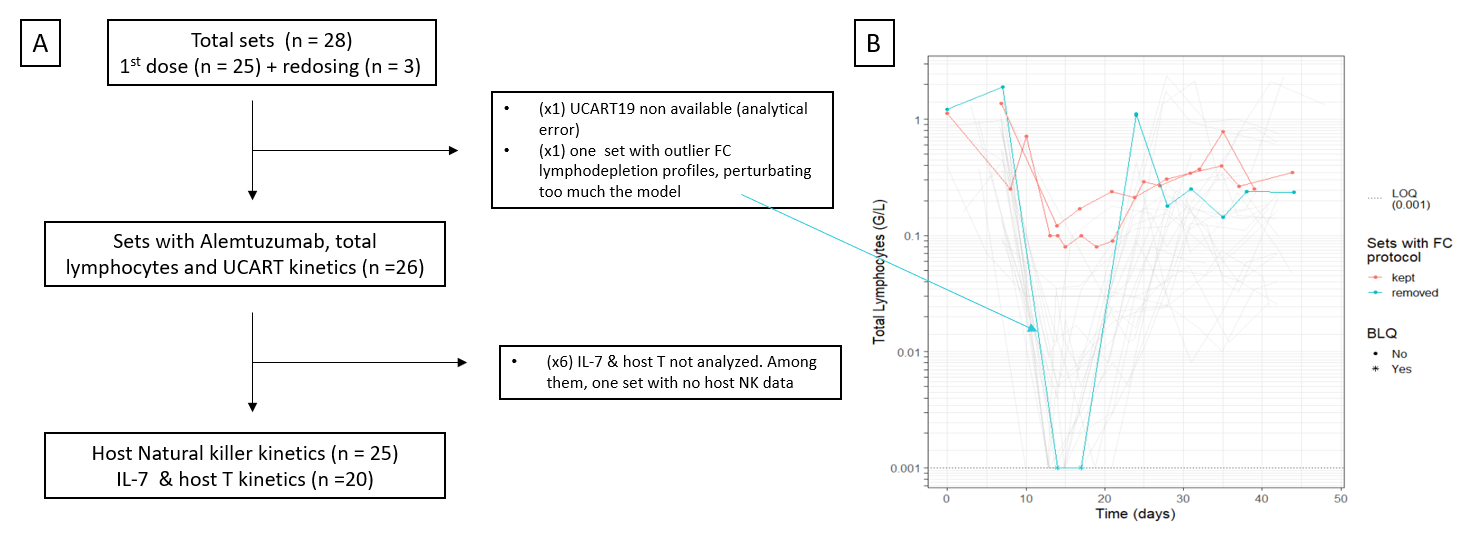


*Supplementary Figure S1: A) Flow of patients: 25 patients received UCART, three of them received two doses. The model considers them as separate patients, such as we have 28 sets. One set was removed for confirmed analytical error, the other one for aberrant values. Out of the 26 remaining sets, six of them do not have IL-7 sampling. B) Total lymphocytes profiles with the highlight of the three sets with FC protocol (background grey profiles are profiles with FCA protocol). Profile in blue was removed from the model as it was considered an outlier profile (abnormal too sharp initial lymphodepletion).*

## Parameters results


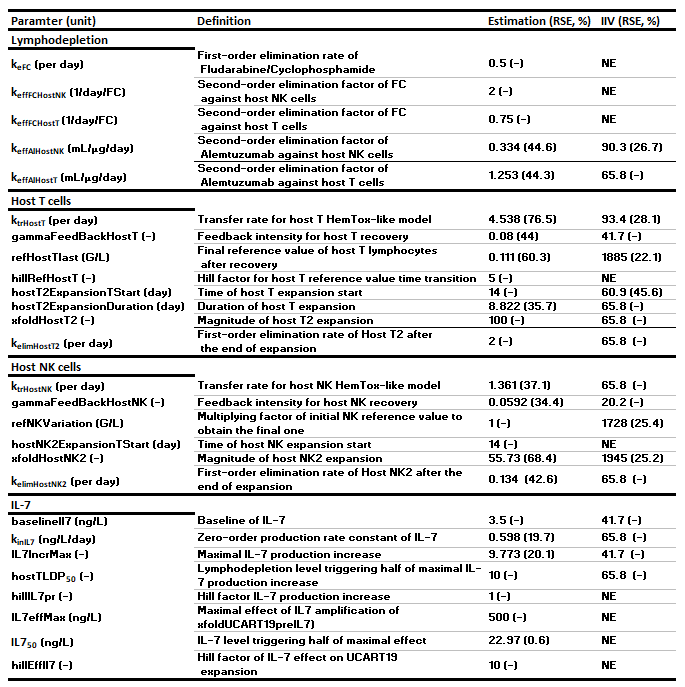


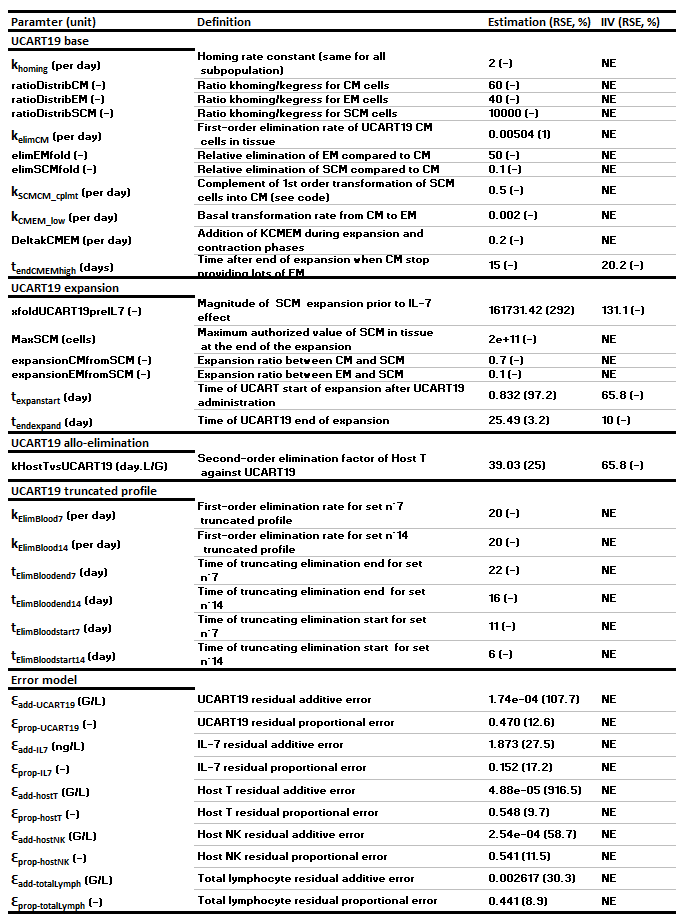
 *Supplementary Table S1: Pharmacokinetic/pharmacodynamic model parameter estimates.*

NOTE:

- Parameter shrinkage values, based on the variance of estimated individual random effects, were greater than 50% for *hostT2ExpansionDuration*, *gammaFeedBackHostNK*, *kelimHostNK2* and *texpanstart*. All other parameters showed shrinkages below 40%–50%.
- Abbreviations: RSE, relative standard error of estimate; IIV, inter-individual variability; NE, not estimated; -, fixed.
- Estimates of IIV are the apparent coefficient of variation for the inter-individual variability (%), using the formula $CV(\%)=\sqrt{(exp(\omega^{2})-1)}\cdot100$, with $\omega$ the standard deviation of the random effects.

## All individual plots


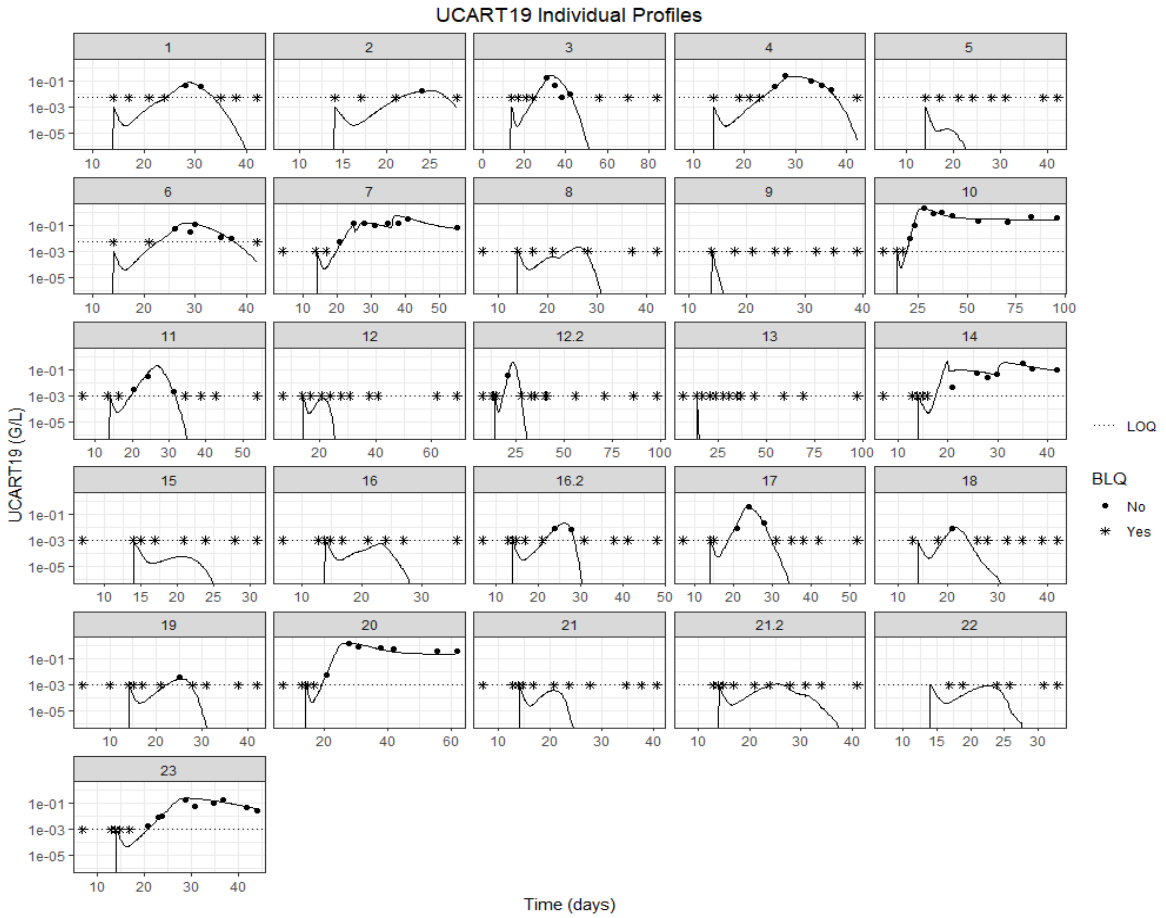


*Supplementary Figure S2A: All UCART19 individual profiles.*


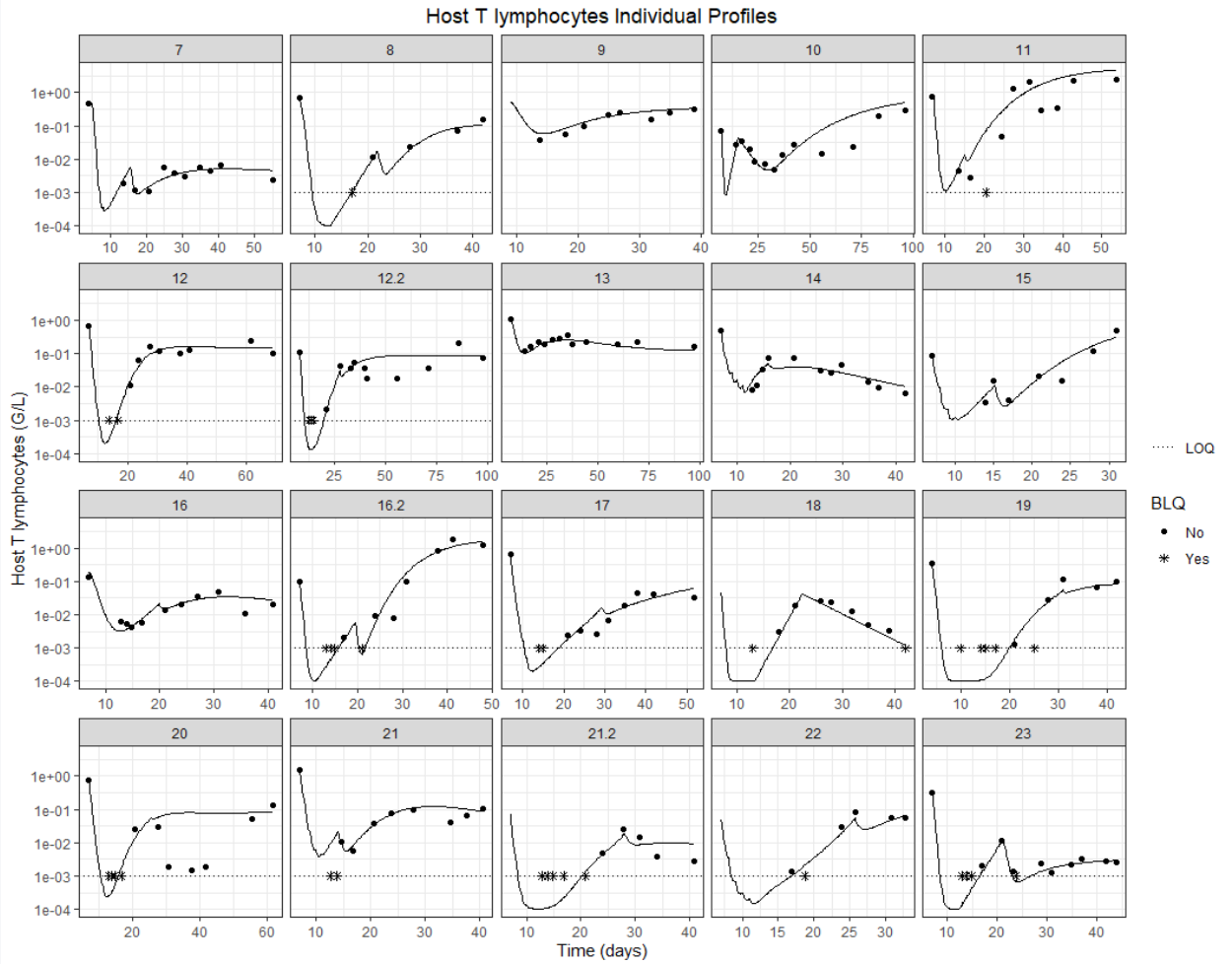


*Supplementary Figure S2B: All host T lymphocytes individual profiles.*


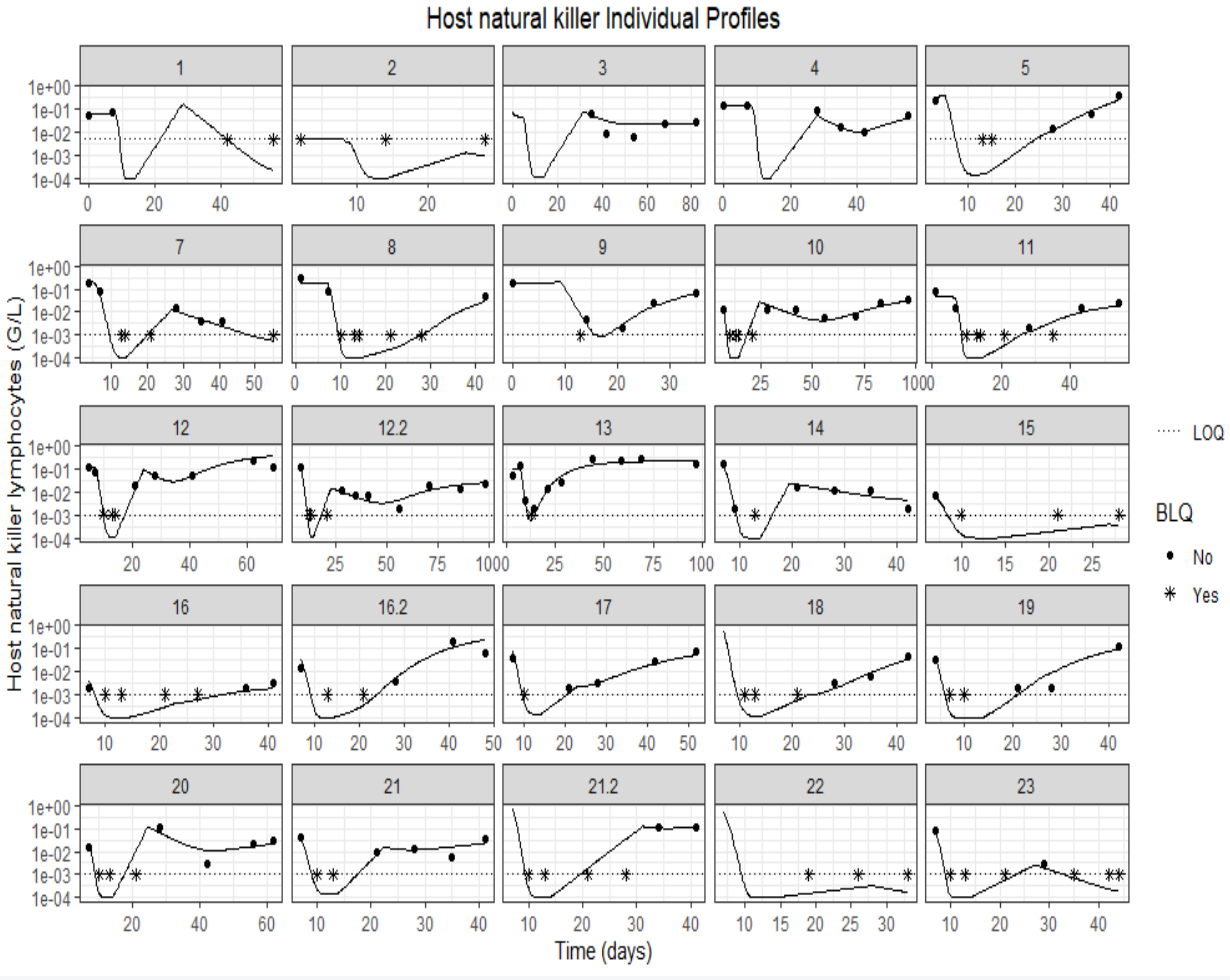


*Supplementary Figure S2C: All host NK lymphocytes individual profiles.*


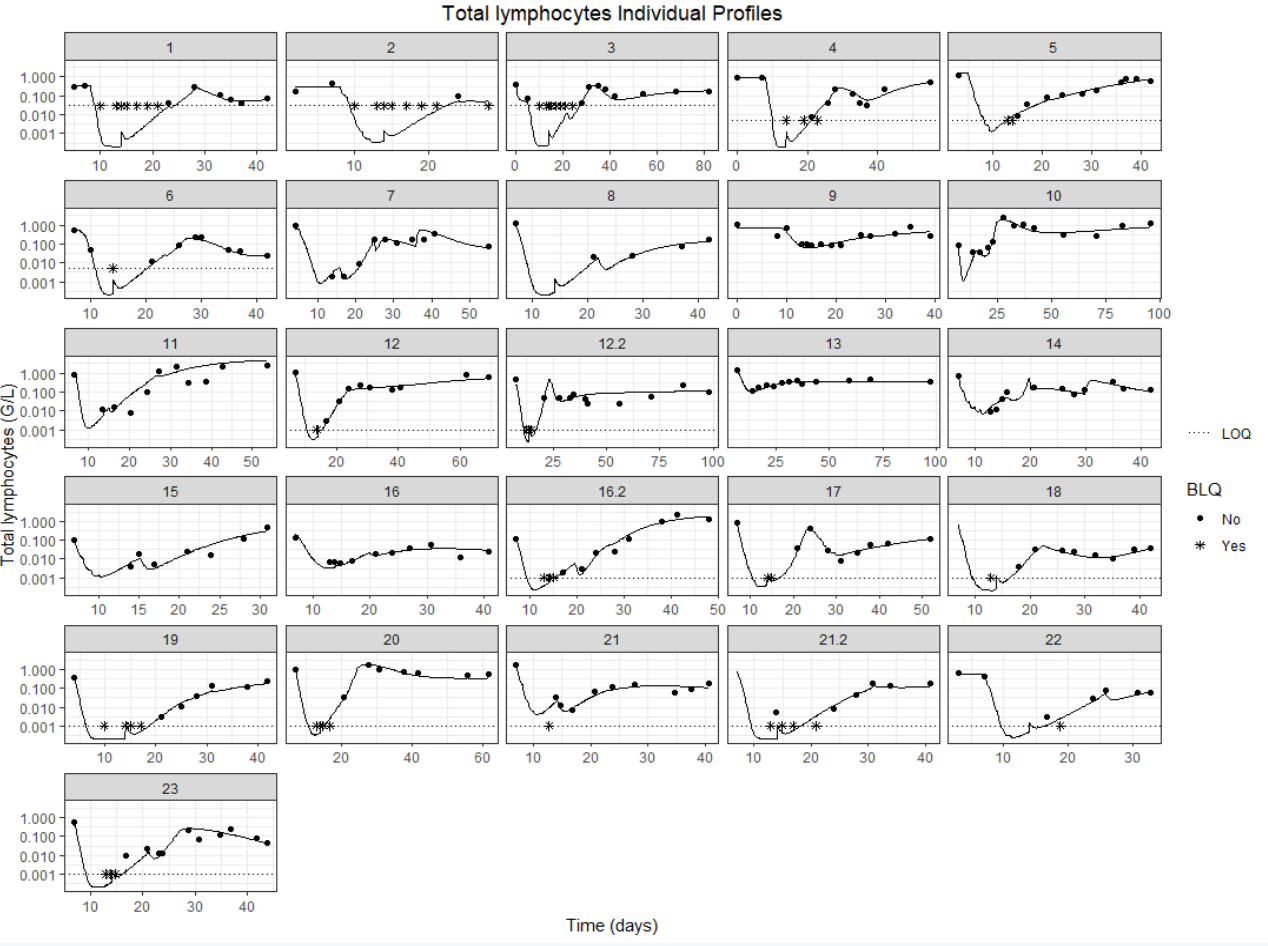


*Supplementary Figure S2D: All total lymphocytes individual profiles.*


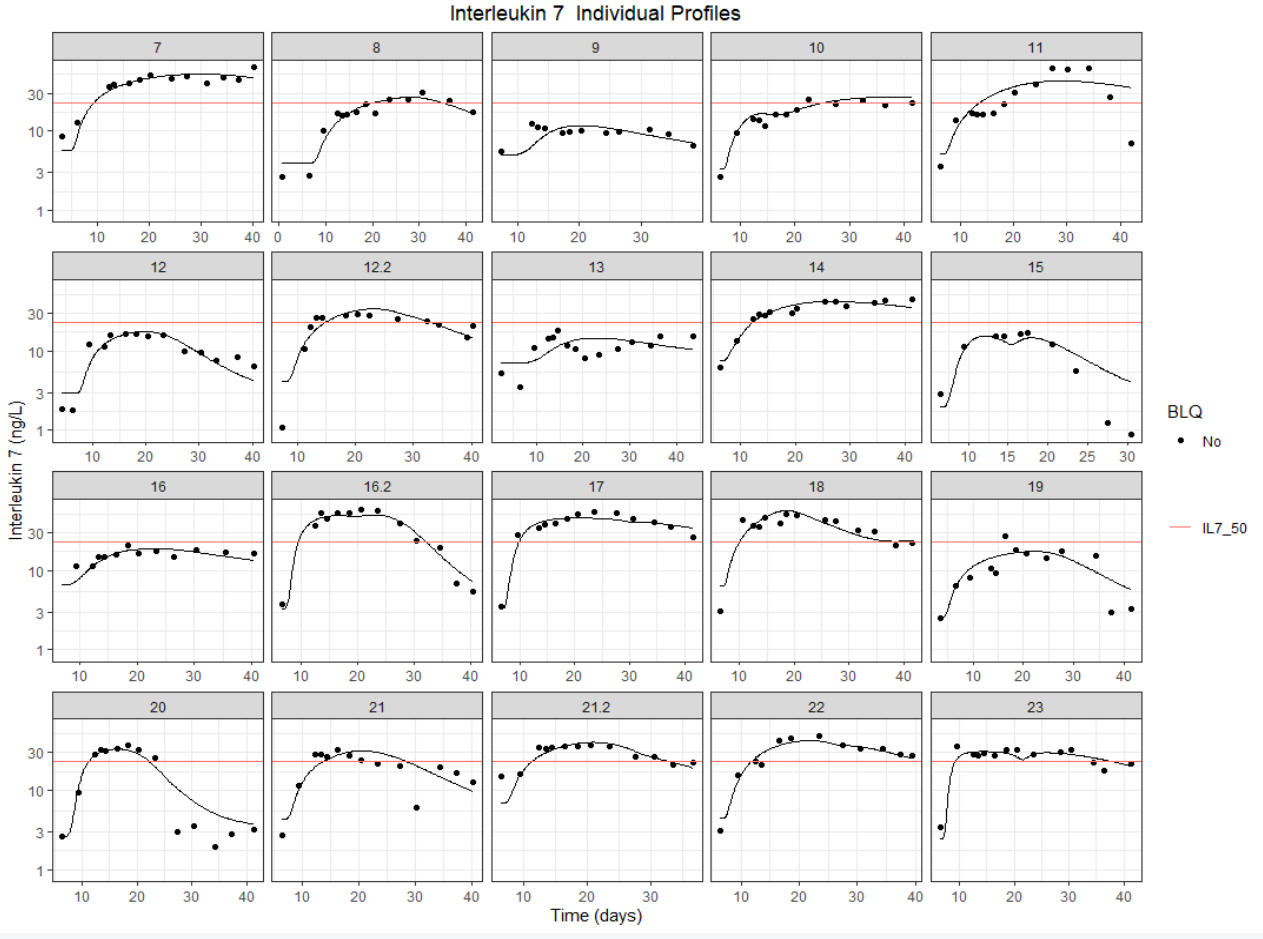


*Supplementary Figure S2E: All IL-7 individual profiles.*

## Goodness of Fit plot


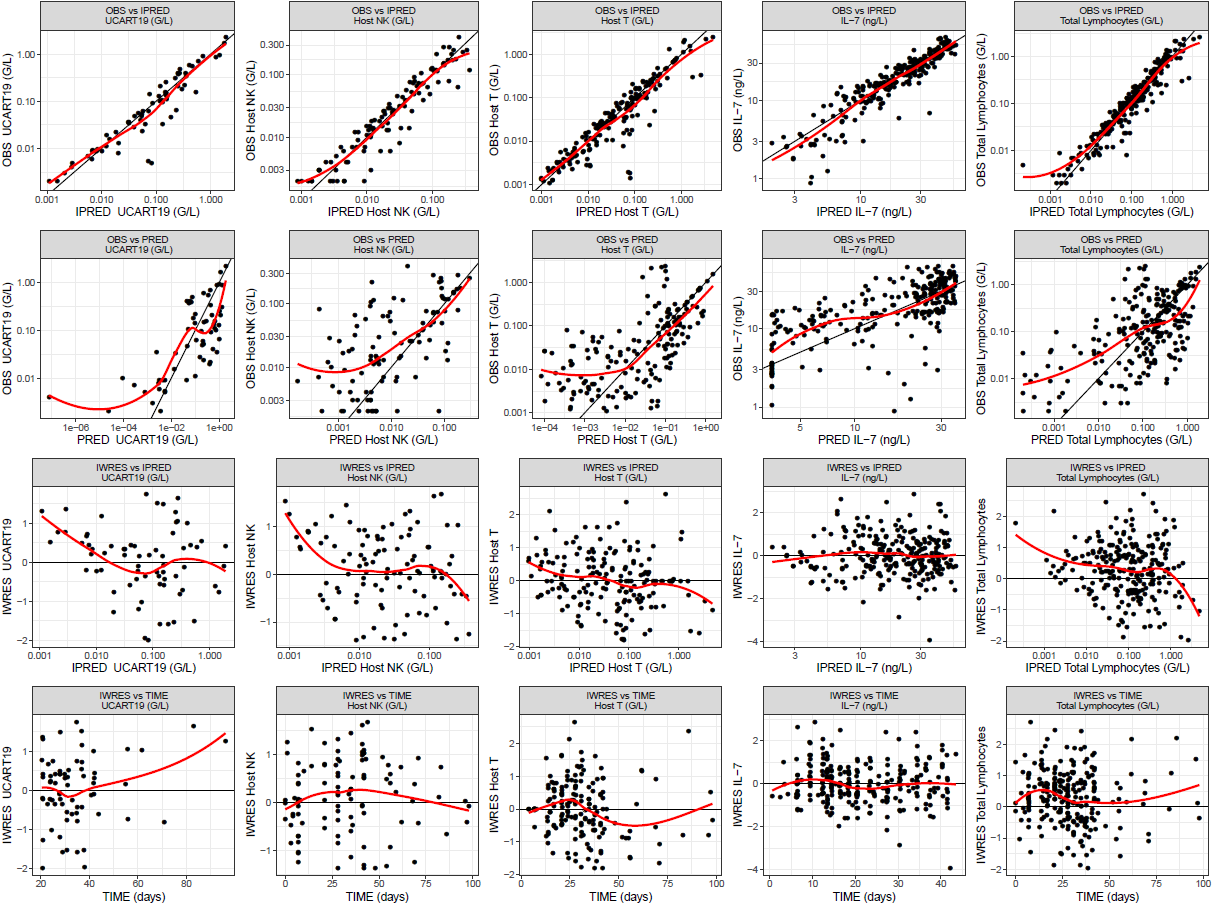


*Supplementary Figure S3: Goodness of Fit plot. Abbreviations: OBS = observation, IPRED = individual prediction, PRED = prediction, IWRES = individual weighted residual. Each column represents an observation type (from left to right: UCART19, host NK, host T, IL-7 and total lymphocytes). Each row represents a specific diagnostic plot (from top to bottom: OBS vs IPRED, OBS vs PRED, IWRES vs IPRED, IWRES vs TIME). Black lines represent identity lines in the two first rows.*

## Alemtuzumab modeling

| Parameter (unit) | Definition | Estimation (RSE, %) | IIV (RSE, %) |
| --- | --- | --- | --- |
| Cl (L/day) | Central clearance | 0.9648 (20.1) | 129.8 (12.1) |
| Q (L/day) | Intercompartmental clearance | 3.513 (61.5) | 3858.1 (12.1) |
| V1 (L ) | Central volume of distribution | 3.746 (13.8) | 47.5 (23) |
| V2 (L) | Peripheral volume of distribution | 6.01 (13.4) | 70.6 (12.1) |
| a (µg/mL) | Additive error | 0.02958 (27.6) | NE |
| b (-) | Proportional error | 0.2022 (7.5) | NE |
| corr_Q_Cl (-) | Covariance between Q and Cl parameters | 0.9027 (NaN) | NE |
| corr_V2_Cl (-) | Covariance between Q and Cl parameters | 0.9748 (NaN) | NE |
| corr_V2_Q (-) | Covariance between Q and Cl parameters | 0.976 (0) | NE |

*Supplementary Table S2: Alemtuzumab pharmacokinetic model parameter estimates.*

NOTE:

- All parameter shrinkage values, based on the variance of estimated individual random effects, were below 40%–50%.
- Abbreviations: RSE, relative standard error of estimate; IIV, inter-individual variability; NE, not estimated; NaN, Not a Number (arithmetic error during computation).
- Estimates of IIV are the apparent coefficient of variation for the inter-individual variability (%), using the formula $CV(\%)=\sqrt{(exp(\omega^{2})-1)}\cdot100$, with $\omega$ the standard deviation of the random effects.


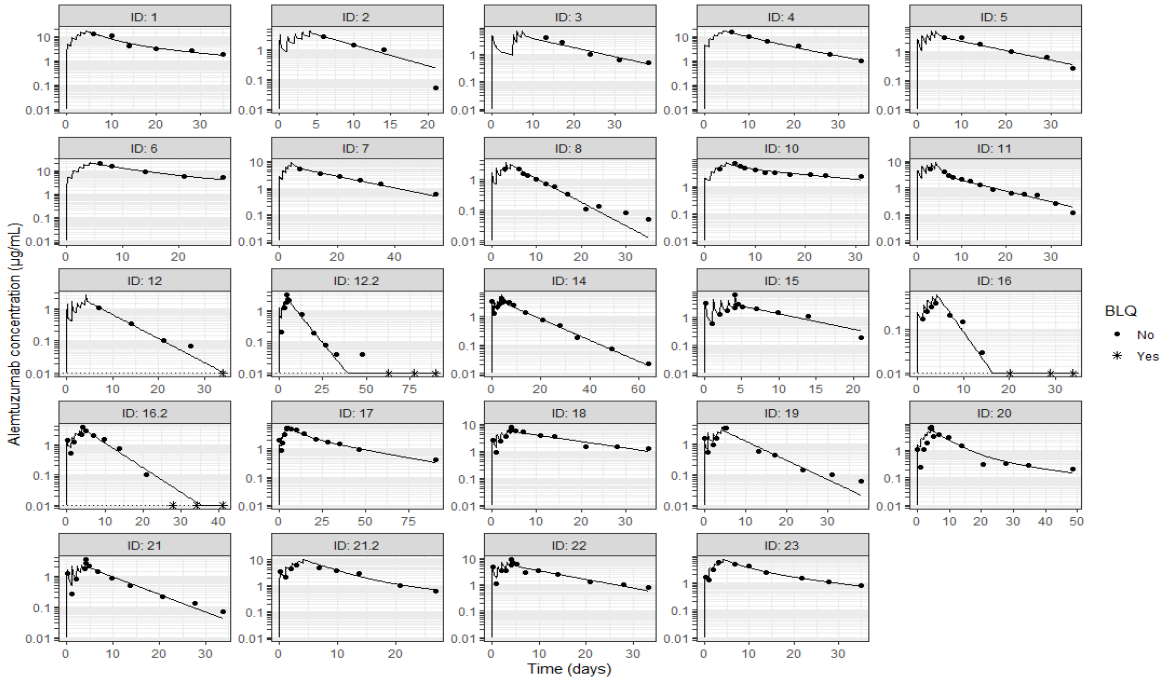


*Supplementary Figure S4A: All alemtuzumab individual prediction.*


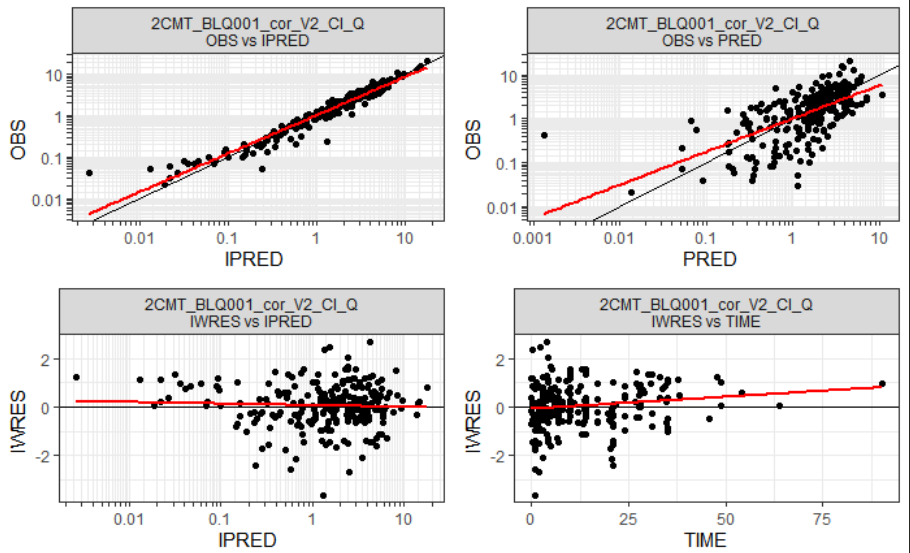


*Supplementary Figure S4B: Goodness of fit plots. Abbreviations: OBS = Observation, PRED = prediction, IPRED = individual prediction, IWRES = individual weighted residual.*


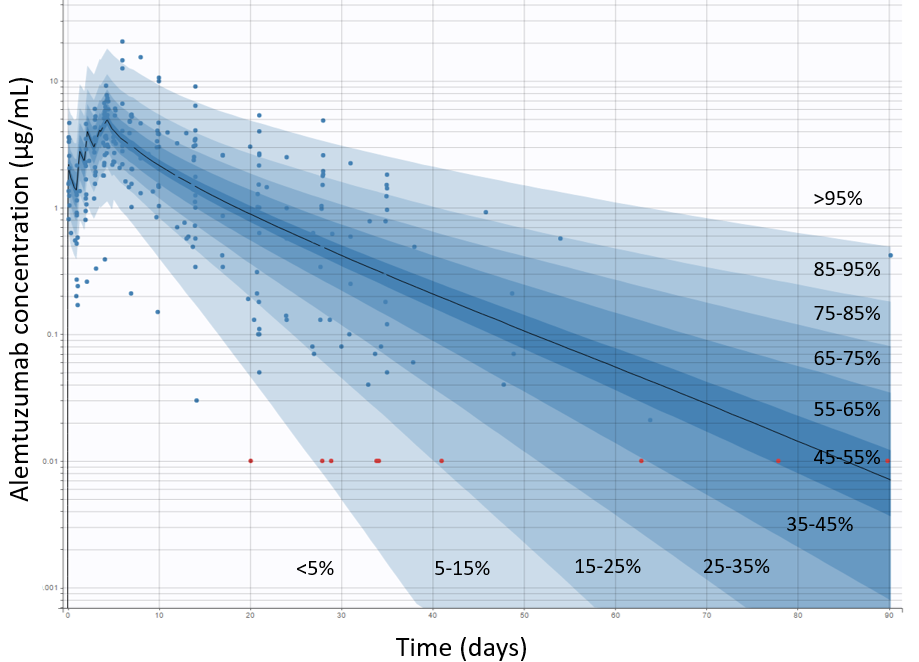


*Supplementary Figure S4C: Prediction distribution plot. Dots represent observations while each band contains ten percent of simulated values.*


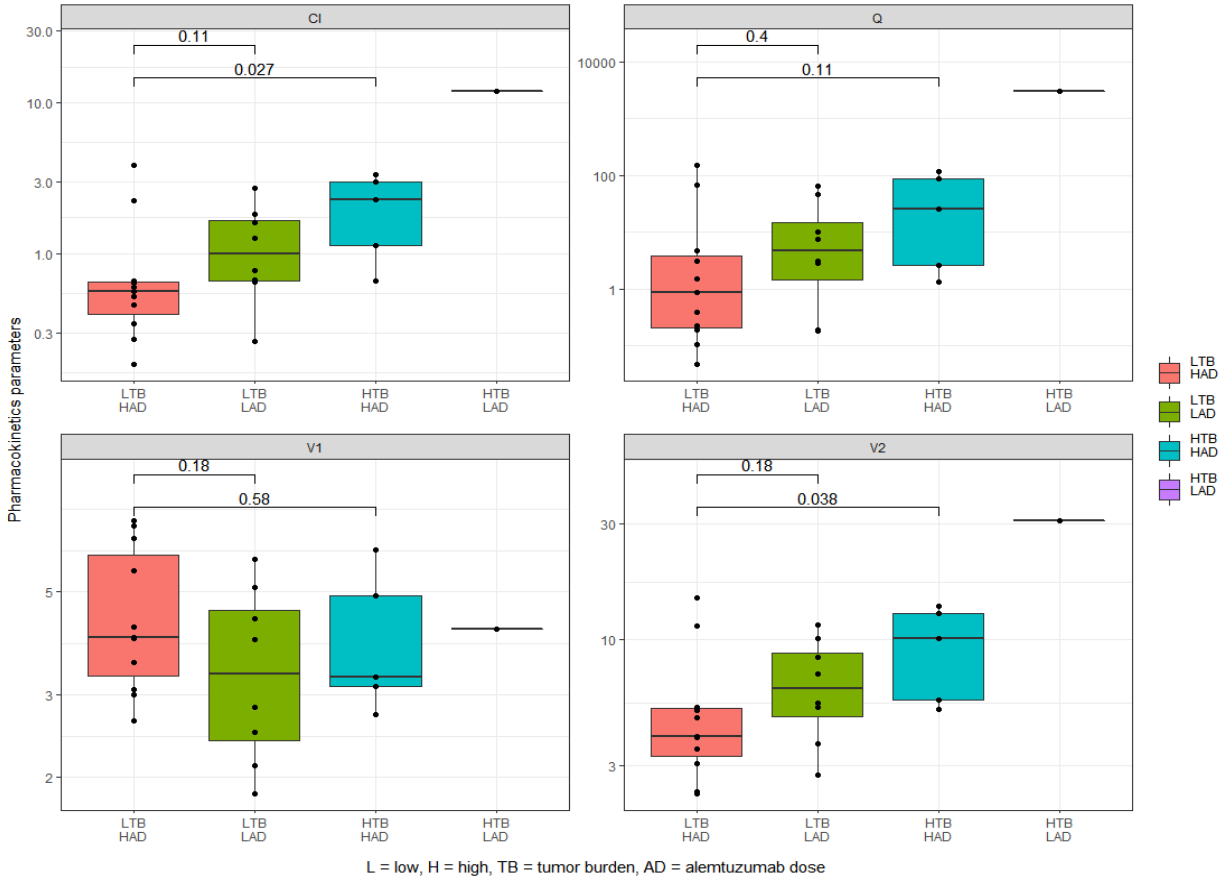


*Supplementary Figure S4D: TMDD highlighting. Parameter individual values have been compared to both alemtuzumab doses (40mg total dose or higher) and tumor burden prior to the administration (below or higher 75%). Both apparent clearance and distribution increase with lowest alemtuzumab doses and highest tumor burden.*

## $\boldsymbol{T}_{\boldsymbol{SCM}}$ and $\boldsymbol{T}_{\boldsymbol{EM}}$/$\boldsymbol{T}_{\boldsymbol{CM}}$ removal


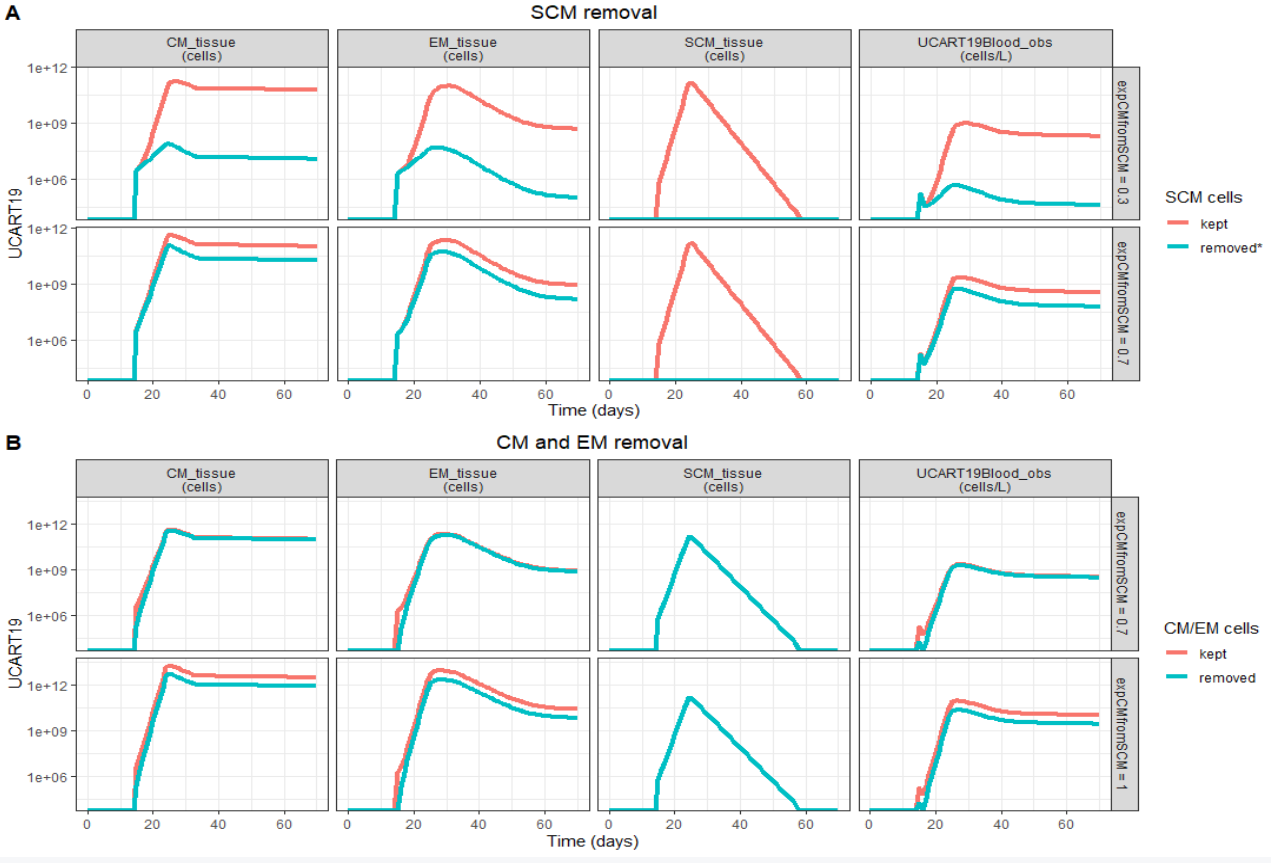


*Supplementary Figure S5: Simulations with no* $T_{SCM}$ *(A) or* $T_{EM}$*/*$T_{CM}$ *(B) injected in the system. Red curves represent the normal UCART19 product (with all types of cells), while blue curves represent simulations with cell removals. A) The impact of* $T_{SCM}$ *removal is strongly dependent on how much* $T_{CM}$ *expand compared to* $T_{SCM}$*. In the second row, where* $T_{CM}$ *expands 0.7-fold quicker than* $T_{SCM}$ *(value used in the main model), removing* $T_{SCM}$ *has a moderate impact on UCART19 expansion. However, when relative expansion has been set to 0.3 (first row) removing* $T_{SCM}$ *leads to the absence of UCART19 observable expansion. B) Removing* $T_{CM}$ *and* $T_{EM}$ *from the product has no to very little impact on UCART19 exposure, as* $T_{SCM}$ *is able to reconstitute every type of cells. Modification of expansionCMfromSCM (set to 0.7 and 1 respectively in first and second rows) does not have an impact on the outcome.*

## Sensitivity analysis


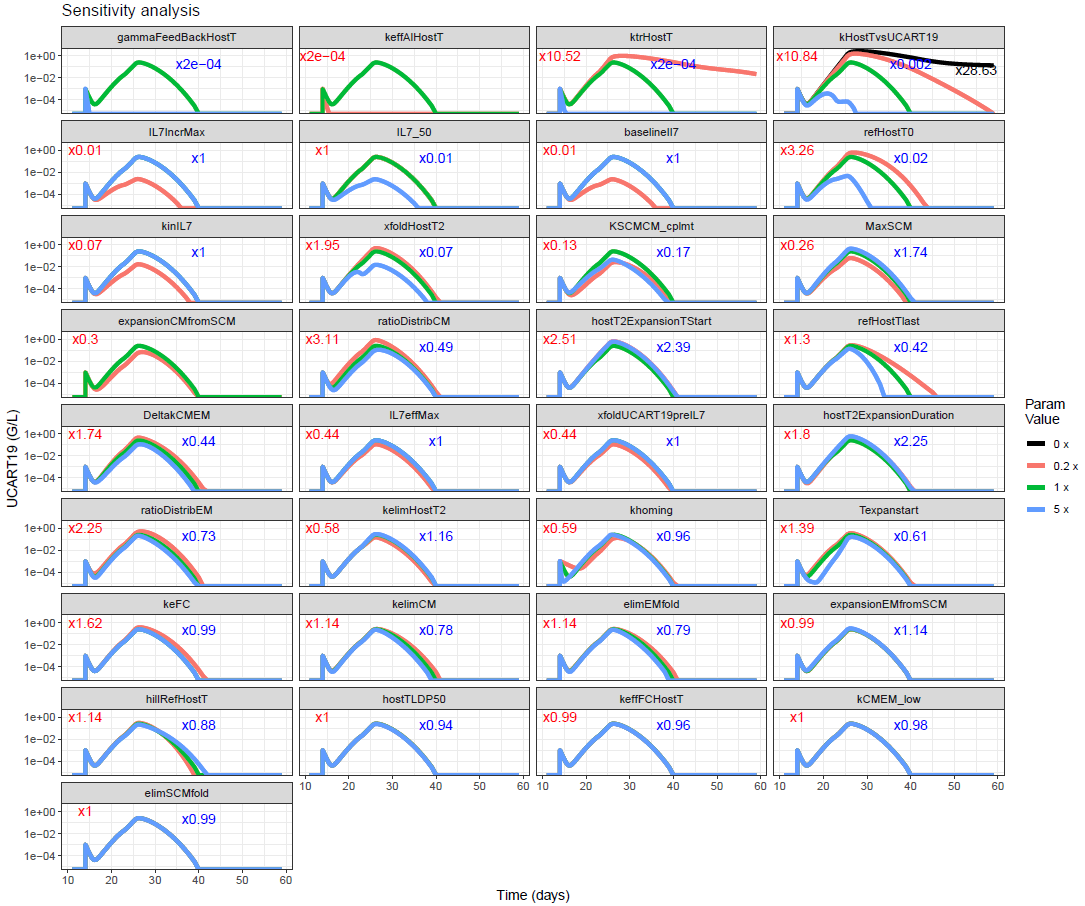


*Supplementary Figure S6: Sensitivity analysis. Each parameter has been multiplied (blue) or divided (red) by 5 from typical value (green), with AUC ratio reported with the same color code. Plots have been ordered from the most to the less impacting parameters. Parameters with no impact have been removed. Some parameter modifications created computational issues and as such are not reported (e.g., keffAlhostT x5).*

## Alternative models

### IL-7 effect

**IL-7 increasing natural growth of UCART19**


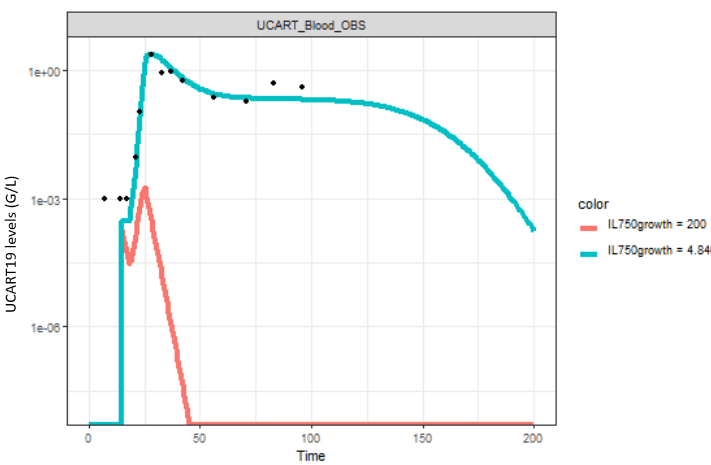


*Supplementary Figure S7A: IL-7 increasing physiological growth of UCART19 model. In blue, a persistence profile in the case of IL-7 levels above the threshold. The profile captured well observed data but dropped after because IL-7 decreased. In red, a profile with IL-7 levels below the threshold with a sharp elimination occurring at every time.*

In this version, $k_{elimX}$ values were higher but counterbalanced partly by a physiological growth $k_{growthX}$. IL-7 played a role directly on $k_{growthX}$ ($k_{growthX}=k_{growthX_{base}}\cdot(1+\frac{IL7growthMax\cdot IL7eff^{\gamma Il_{7}}}{IL7eff^{\gamma Il_{7}}+IL750growth^{\gamma Il_{7}}})$). The role of IL-7 was thus to maintain the ratio $k_{growthX}-k_{elimX}$ close to 0 in order to avoid early elimination and create the persistence slope. It was actually able to capture our profile but, when prolonging the simulation, the profiles dropped when IL-7 returned below to IL750. In this example, the model adapted this individual IL750 with a very low value, such as the persistence slope is maintained up to the observations.

**IL-7 inhibiting an initial engraftment elimination**


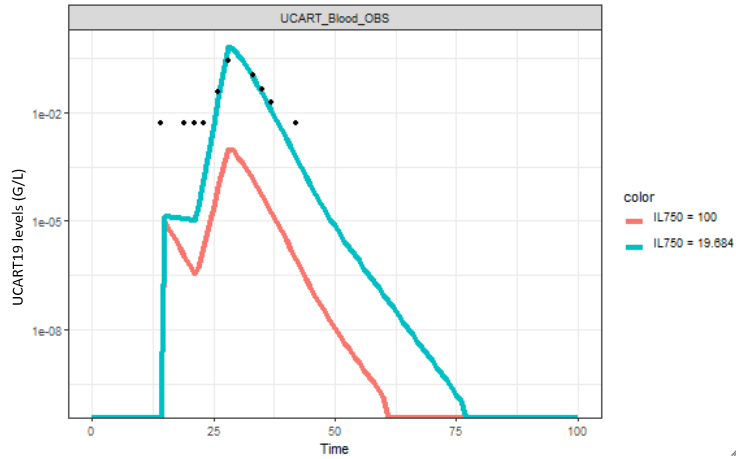


*Supplementary Figure S7B: early rejection of engrafted UCART19 cells alternative model. In this version, UCART19 undergoes an additional elimination occurring until the end of UCART19 expansion. This elimination is inhibited by IL-7. In red, a profile with Il-7 levels below the threshold. In blue, a profile with this elimination being inhibited by IL-7. The last elimination slope, after the expansion is due to the allogeneic elimination.*

### Double slope

$\boldsymbol{T}_{\boldsymbol{CM}}$**becoming** $\boldsymbol{T}_{\boldsymbol{EM}}$


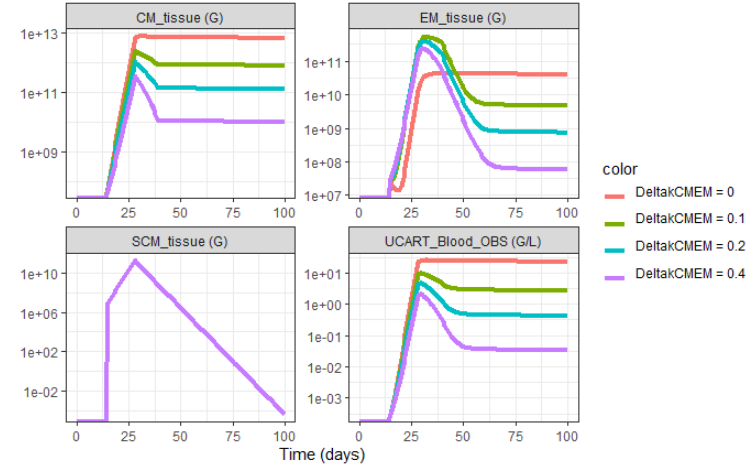


*Supplementary Figure S8: Impact of deltakCMEM*

In this version (the one described in the main paper), $\Delta k_{CMEM}$ allow to describe, during a period of time, an increased transformation from $T_{CM}$ to $T_{EM}$, which will last some days after the UCART expansions. In this version. The $\Delta k_{CMEM}$ control the slope, but as it has an impact since the beginning of the expansion, it will also impact the Cmax.

**Modifying egress**


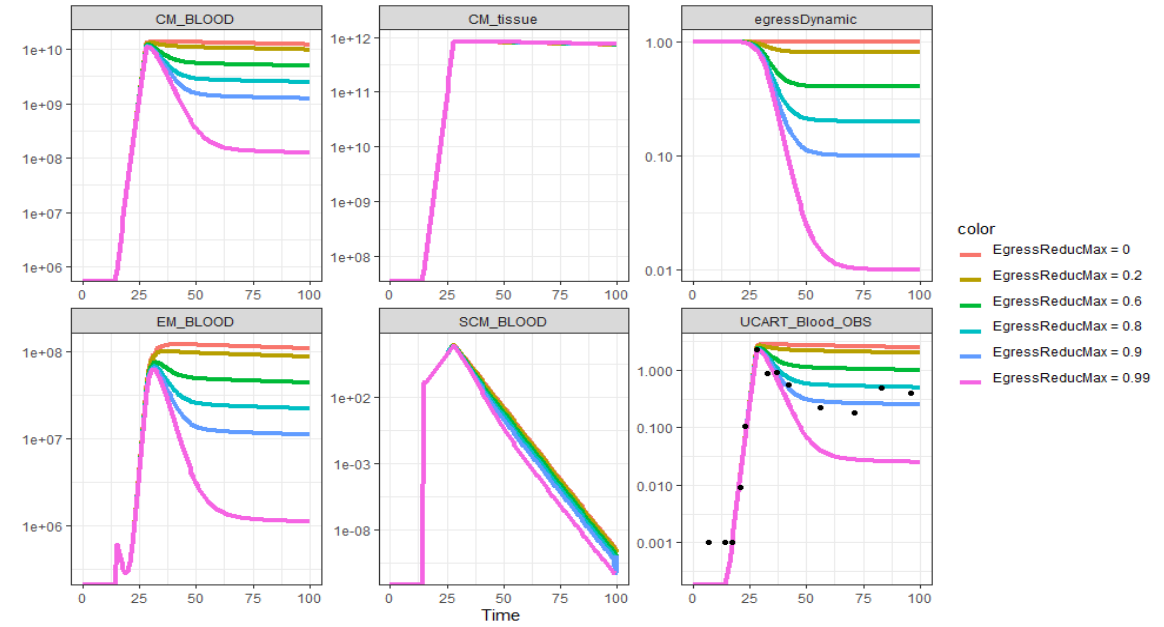


*Supplementary Figure S9: Modifying homing to create contraction phase. See below for explanations.*

In this alternative version, a modification of egress creates the slope. Previously fixed $k_{egressX}$ are multiplied by $egressDynamic(t)=1-\frac{EgressReducMax\cdot t^{\gamma_{eg}}}{t^{\gamma_{eg}}+(T_{endExp}+t_{50egress})^{\gamma_{eg}}}$ , with $T_{endExp}$ the time of expansion ending).

In these simulations, we first set $\Delta k_{CMEM}$ to 0. $\gamma_{eg}=3$ and $t_{50egress}=5$ were set after manual selection. Here, progressive egress reduction with a final egress divided by 10 allows to capture well the contraction and persistence phase. $T_{CM}$ and $T_{EM}$ do follow the same dynamics.

**Exhaustion, AICD...**


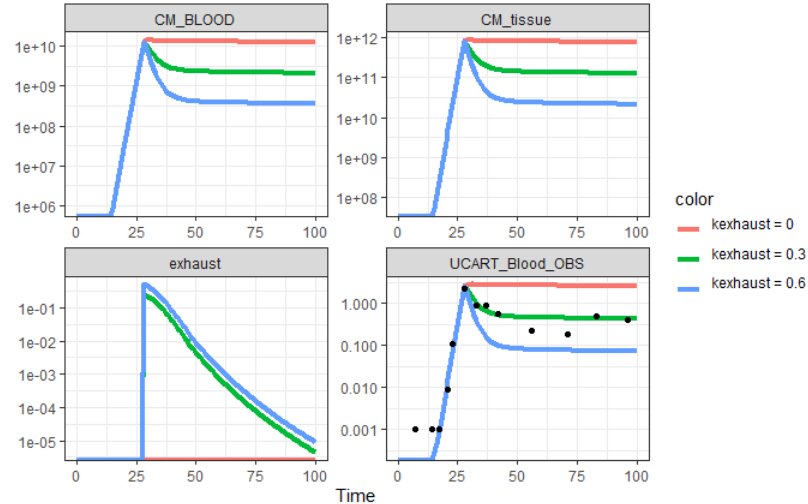


*Supplementary Figure S10: Adding elimination to create contraction phase. See below for explanations.*

In this alternative version, a new elimination starts at the end of the expansion and then decrease under an Emax model. This elimination is applied to each compartment. It might be seen as exhaustion or AICD death. $T_{CM}$ and $T_{EM}$ follow the same dynamics.

- $exhaust=0$ before the end of expansion ($t<T_{endExp}$)
- $exhaust=k_{exhaust}\cdot(1-\frac{t^{\gamma_{ex}}}{(t^{\gamma_{ex}}+(T_{endExp}+t50ex)^{\gamma_{ex}})})$ , with $T_{endExp}$ the time of expansion ending).

In these simulations, we first set $\Delta k_{CMEM}=0$, plus $\gamma_{ex}=3$ and $t_{50ex}=5$ after manual selection.

## Elimination in blood


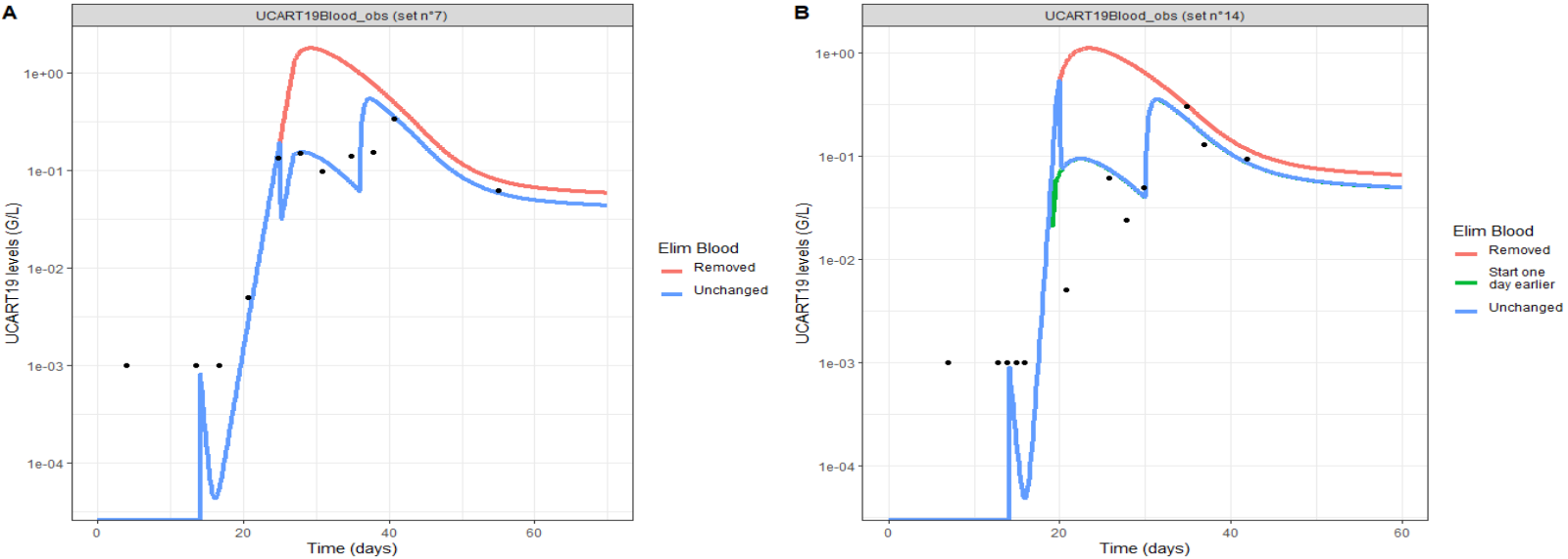


*Supplementary Figure S11: In blue, the two UCART19 profiles undergoing the elimination in blood. In red, simulation of the same profiles without the elimination. In green (B) the same elimination is triggered one day earlier to highlight the first decline (before a new equilibrium between homing and egress).*

## Potential use of the model to optimize CAR-T cells therapies


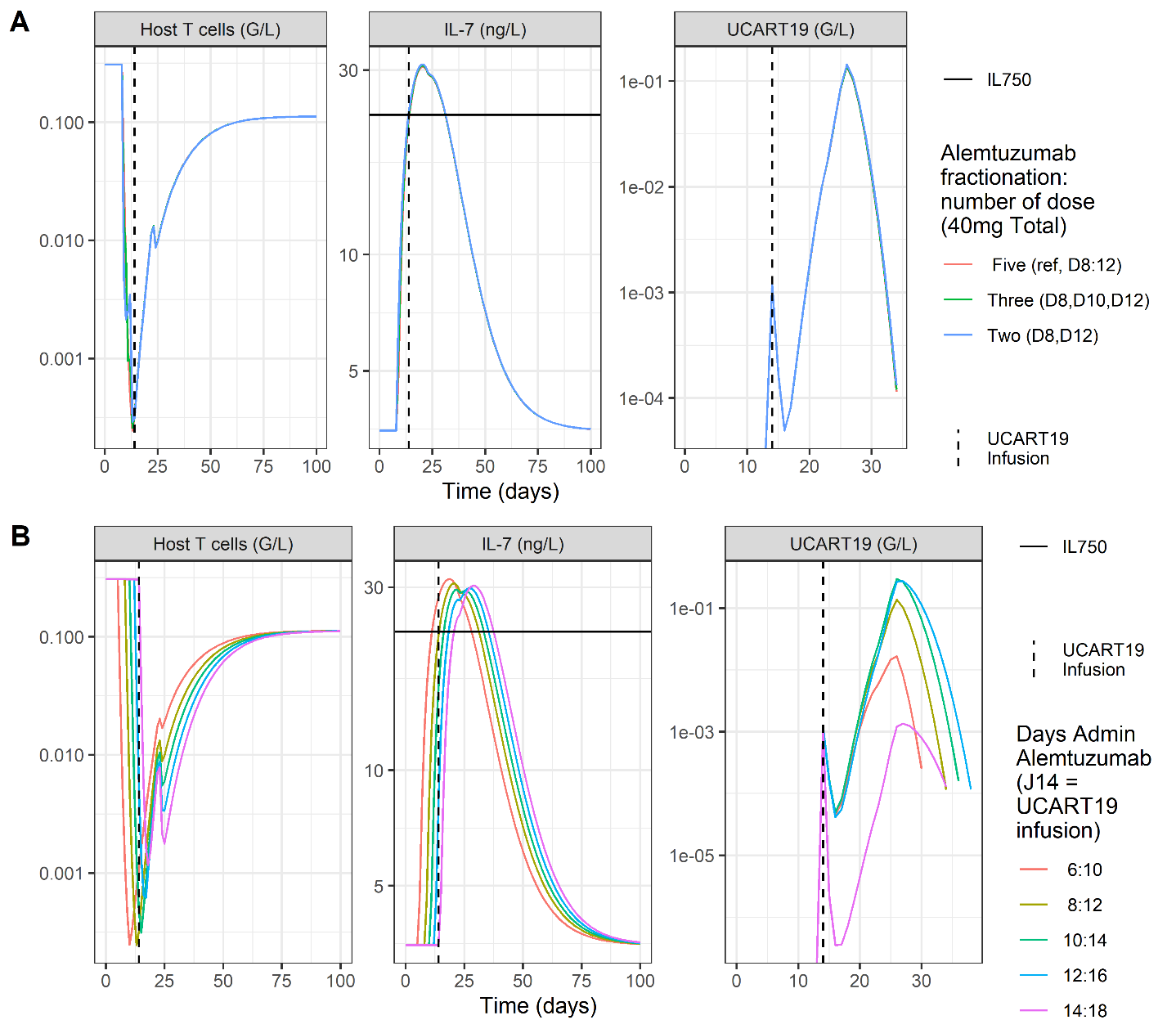


*Supplementary Figure S12: Potential application of the model to optimize lymphodepleting regimen through simulation exercises. The reference profiles correspond to a patient with typical values for all parameters, a total dose of 40mg of Alemtuzumab, and an infusion of 6E6 UCART19 cells (equally distributed among* $T_{SCM}$*,* $T_{CM}$*, and* $T_{EM}$*). FC protocol was inputted on the first day of Alemtuzumab administration. Each row corresponds to a different set of dose regimen modifications, and columns represent host T cells, IL-7 and UCART19 levels. A) Simulations with different fractionation of 40mg of Alemtuzumab (2x20mg, 3x13mg, 5*8mg) led to unchanged results. B) Impact of the starting date of the 5-day lymphodepletion regimen. The lymphodepletion should start neither too early (red) nor too late (purple). Regardless of any clinical consideration, the most effective regimen for this patient would be to start the lymphodepletion two days before UCART19 infusion and to continue for 2 days post UCART19 infusion (blue).*

## Monolix code

; Date: 01/14/2021
; Thibaud Derippe

[LONGITUDINAL]

input ={
; ----- Alemtuzumab related input/parameters
CL, V1, Q, V2, keffAlHostT, keffAlHostNK,

; ----- Fludarabine / Cyclophosphamide related parameters
 keFC, keffFCHostT, keffFCHostNK,

; ----- Host T classic recovery system related parameters
refHostT0, refHostTlast , hillRefHostT, gammaFeedBackHostT, ktrHostT, kHostTvsUCART19 ,

; ----- Host T peak system related parameters
 hostT2ExpansionTStart, hostT2ExpansionDuration, xfoldHostT2, kelimHostT2,

; ----- Host NK classic recovery system related parameters
refHostNK0, refNKVariation, gammaFeedBackHostNK, ktrHostNK,

; ----- Host NK peak system related parameters
 hostNK2ExpansionTStart, xfoldHostNK2, kelimHostNK2,

; ----- Interleukin 7 related parameters
 kinIL7, baselineIl7, IL7IncrMax , hostTLDP50, hillIL7pr, IL7_50, hillEffIl7,

; ---- UCART 19 related parameters
 tendexpand, texpanstart, expansionCMfromSCM , expansionEMfromSCM, kelimCM, elimSCMfold, elimEMfold, kelimgraft, khoming,ratioDistribSCM ,ratioDistribCM, ratioDistribEM , kSCMCM_cplmt, MaxSCM, xfoldUCART19preIL7,IL7effMax , kCMEM_low, DeltakCMEM , tElimBloodstart7, tElimBloodend7, kElimBlood7, tElimBloodstart14, tElimBloodend14, kElimBlood14,tendCMEMhigh,

; ---- additional regressors
autologousLikeID, IDcov

}


; ###################################### Regressors declaration ########################

CL = {use=regressor}
V1 = {use=regressor}
Q = {use=regressor}
V2 = {use=regressor}
autologousLikeID = {use=regressor}
refHostNK0 = {use=regressor}
refHostT0 = {use=regressor}
IDcov = {use=regressor}

PK:

 ; ###################################### Administrations handling ########################

; --- Administration Alemtuzumab, perfusion 2h (= 0.0833 days) ---
 depot(target = AlCentral, adm = 1, Tk0 = 0.08333)

; --- Administration Fludarabine/Cyclophosphamide (KPD context), Intravenous
depot(target = FC, adm = 2)

; --- Administration UCART19 in blood, per subpopulation
depot(target = SCM_BLOOD, adm = 3)
depot(target = CM_BLOOD, adm = 4)
depot(target = EM_BLOOD, adm = 5)

; --- Copie of SCM_BlOOD compartment (will be frozen, see after)
depot(target = SCM_BLOOD2, adm = 3)


EQUATION:


 ; ###################################### Initial Conditions ########################
t0 = 0

AlCentral_0 = 0
AlPeriph_0 = 0
FC_0 = 0

HostT1TISSUE_0 = refHostT0
HostT1Blood_0 = refHostT0
HostT2_0 = 0.0001

HostNK1TISSUE_0 = refHostNK0
HostNK1Blood_0 = refHostNK0
HostNK2_0 = 0.0001

IL7_0 = baselineIl7

SCM_TISSUE_0 = 0
SCM_BLOOD_0 = 0
CM_TISSUE_0 = 0
CM_BLOOD_0 = 0
EM_TISSUE_0 = 0
EM_BLOOD_0 = 0

SCM_TISSUE2_0 = 0
SCM_BLOOD2_0 = 0

; ###################################### Lymphodepletion Pharmacokinetics ########################

;---------- PK Alemtuzumab -------------

 KE = CL / V1
K12 = Q / V1
K21 = Q / V2

ddt_AlCentral = - AlCentral * (K12 + KE) + AlPeriph * K21
ddt_AlPeriph = - AlPeriph * K21 + AlCentral * K12
Alemtuzumab = AlCentral/ V1

;------------ Virtual PK FC --------------------

 ddt_FC = -FC * keFC


; ###################################### Host T Lymphocytes ########################

; -- Host T baseline handling (changing after UCART19 end expanding)

deltaRefHostT = refHostT0 - refHostTlast

refHostT = refHostT0 - deltaRefHostT * t ^ hillRefHostT / (tendexpand ^ hillRefHostT + t ^ hillRefHostT )


; -- Host T computation classic recovery system

feedbackHostT = (refHostT / (HostT1Blood)) ^ gammaFeedBackHostT

ddt_HostT1TISSUE = HostT1TISSUE * (ktrHostT * feedbackHostT - ktrHostT - Alemtuzumab * keffAlHostT- FC * keffFCHostT)
ddt_HostT1Blood = HostT1TISSUE * ktrHostT - HostT1Blood * (ktrHostT + Alemtuzumab * keffAlHostT)

; -- Host T expansion

;- time handling
if t < hostT2ExpansionTStart ; (will be fixed to 14 - time of UCART19 infusion - with normal distribution)
 bool_expansionHostT2 = 0
 bool_elimHostT2 = 0
elseif t < hostT2ExpansionTStart + hostT2ExpansionDuration
 bool_expansionHostT2 = 1
 bool_elimHostT2 = 0
else
 bool_expansionHostT2 = 0
 bool_elimHostT2 = 1
end


ddt_HostT2 = HostT2 * (bool_expansionHostT2 * log(xfoldHostT2) / hostT2ExpansionDuration - kelimHostT2 * bool_elimHostT2)


; -- Final host T lymphocytes computation

HostT_obs = HostT1Blood + HostT2


; ###################################### Host Natural killers ########################

; -- Host NK baseline handling (changing after UCART19 end expanding)

deltaRefHostNK = refHostNK0 * refNKVariation - refHostNK0

refHostNK = refHostNK0 + deltaRefHostNK * t / (tendexpand + t )


; -- Host NK computation classic recovery system

feedbackHostNK = (refHostNK / (HostNK1Blood)) ^ gammaFeedBackHostNK

ddt_HostNK1TISSUE = HostNK1TISSUE * (ktrHostNK * feedbackHostNK - ktrHostNK - Alemtuzumab * keffAlHostNK- FC * keffFCHostNK)
ddt_HostNK1Blood = HostNK1TISSUE * ktrHostNK - HostNK1Blood * (ktrHostNK + Alemtuzumab * keffAlHostNK)

; -- Host NK expansion

;- expansion time handling
if t < hostNK2ExpansionTStart ; (will be fixed to 14 - time of UCART19 infusion - with normal distribution)
 bool_expansionHostNK2 = 0
 bool_elimHostNK2 = 0
elseif t < tendexpand
 bool_expansionHostNK2 = 1
 bool_elimHostNK2 = 0
else
 bool_expansionHostNK2 = 0
 bool_elimHostNK2 = 1
end


ddt_HostNK2 = HostNK2 * (bool_expansionHostNK2 * log(xfoldHostNK2) / (tendexpand - hostNK2ExpansionTStart) - kelimHostNK2 * bool_elimHostNK2)


; -- Final Host NK lymphocytes computation

HostNK_obs = HostNK1Blood + HostNK2


; ###################################### Interleukin 7 ########################

hostTLDP = refHostT / HostT_obs


if hostTLDP > 1
increaseIL7 = IL7IncrMax * (hostTLDP - 1) ^ hillIL7pr / ( (hostTLDP - 1) ^ hillIL7pr + hostTLDP50 ^ hillIL7pr)
else
 increaseIL7 = 0
end

; note: the ifelse structure was made in case of the peak HostT2 is too high (leading to hostTRatioFromBsl < 1), which in the end create computational issues

koutIL7 = kinIL7 / baselineIl7
ddt_IL7 = kinIL7 * (1 + increaseIL7) - koutIL7 * IL7


; ###################################### UCART19 ########################


durationexpansion = tendexpand - texpanstart - 14

;-- UCART time handling
 if t < 14 + texpanstart
 bool_UCARTexpansion = 0
 bool_SCM2 = 1
elseif t < tendexpand
 bool_UCARTexpansion = 1
 bool_SCM2 = 0
else
 bool_UCARTexpansion= 0
 bool_SCM2 = 0
end


; - eggress by subtypes (constant homing) -

kegress_SCM = khoming / ratioDistribSCM
kegress_CM = khoming / ratioDistribCM
kegress_EM = khoming / ratioDistribEM

; -- elim --
kelimSCM = kelimCM * elimSCMfold
kelimEM = kelimCM * elimEMfold ; elimEM is higher than CM to create contraction and persistance phases


; - SCM to CM transformation -
kSCMCM = kelimCM * ( 1 - elimSCMfold) + kSCMCM_cplmt

; - double slope by controling KCMEM -

if t < 14 + texpanstart
kCMEM = kCMEM_low
elseif t < 14 + texpanstart + durationexpansion + tendCMEMhigh
kCMEM = kCMEM_low + DeltakCMEM
else
kCMEM = kCMEM_low
end


; - computing the expansion rate of UCART19 with a safeguard system (to avoid too high values) -
; explanation: if we don't have this system, xfoldUCART19Maxcan reach, especially during simulations, value too high which will result in non-physiological UCART19 values. This system analyses the SCM_TISSUE value at the end of the UCART19 expansion, and, if needed, reduce the expansion rate accordingly to reach the MaxSCM value.

kexpanbase = log(xfoldUCART19preIL7 * (1 + IL7effMax * IL7 ^ hillEffIl7 / (IL7 ^ hillEffIl7 + IL7_50 ^ hillEffIl7) ) ) / durationexpansion

rateTISSUESCM = kexpanbase - kelimSCM - kSCMCM ; here is the sum of all SCM tarnsfert

valueSCMendExpansion = SCM_TISSUE2 * exp(rateTISSUESCM * durationexpansion) ; here is the value SCM will have at the end of the expansion

if valueSCMendExpansion > MaxSCM
kexpan = log(MaxSCM / SCM_TISSUE2 ) / durationexpansion + kelimSCM + kSCMCM
else
kexpan = kexpanbase
end


if SCM_TISSUE2 == 0 ; - just to avoid computational issue (before UCART19 administration, MaxSCM / SCM_TISSUE2 cannot be computed)
expansionSCM = 0
else
expansionSCM = bool_UCARTexpansion * kexpan
end


expansionCM = expansionSCM * expansionCMfromSCM
expansionEM = expansionSCM * expansionEMfromSCM


; - ElimBlood -

if IDcov == 7
tElimBloodstart = 14 + tElimBloodstart7
tElimBloodend = 14 + tElimBloodend7
kElimBlood = kElimBlood7
elseif IDcov == 14
tElimBloodstart = 14 + tElimBloodstart14
tElimBloodend = 14 + tElimBloodend14
kElimBlood = kElimBlood14
else
tElimBloodstart = 0
tElimBloodend = 0
kElimBlood = 0
end


if t < tElimBloodstart
ElimBloodbool = 0
elseif t < tElimBloodend
ElimBloodbool = 1
else
ElimBloodbool = 0
end

; - elimination
elimallo = HostT_obs * kHostTvsUCART19 * (1 - autologousLikeID);autologousLikeID

; - main equations -


ddt_SCM_TISSUE = SCM_TISSUE * (expansionSCM - kelimSCM - kegress_SCM - kSCMCM) + khoming * SCM_BLOOD - SCM_TISSUE * elimallo
ddt_SCM_BLOOD = kegress_SCM * SCM_TISSUE - khoming * SCM_BLOOD - ElimBloodbool * kElimBlood * SCM_BLOOD - SCM_BLOOD * elimallo


ddt_CM_TISSUE = CM_TISSUE * (expansionCM - kelimCM - kegress_CM - kCMEM) + CM_BLOOD * khoming + SCM_TISSUE * kSCMCM - CM_TISSUE *elimallo
ddt_CM_BLOOD = CM_TISSUE * kegress_CM - CM_BLOOD * khoming - ElimBloodbool * kElimBlood * CM_BLOOD - CM_BLOOD * elimallo

ddt_EM_TISSUE = EM_TISSUE * (expansionEM - kegress_EM - kelimEM ) + EM_BLOOD * khoming + CM_TISSUE * kCMEM - EM_TISSUE * elimallo
ddt_EM_BLOOD = kegress_EM * EM_TISSUE - khoming * EM_BLOOD - ElimBloodbool * kElimBlood * EM_BLOOD - EM_BLOOD * elimallo

;-- freeze compartment SCM to get value before expansion --

ddt_SCM_TISSUE2 = (SCM_TISSUE2 * ( kelimSCM - kegress_SCM - kSCMCM) + khoming * SCM_BLOOD2 - SCM_TISSUE * elimallo) * bool_SCM2
ddt_SCM_BLOOD2 = (kegress_SCM * SCM_TISSUE2 - khoming * SCM_BLOOD2 - SCM_BLOOD * elimallo) * bool_SCM2


; ###################################### Final output ########################

UCART19Blood_obs = (CM_BLOOD + EM_BLOOD + SCM_BLOOD) / 5e+09 ; (transformation in G/L, assuming 5L of blood)
LymphTotal_obs = HostT_obs + HostNK_obs + UCART19Blood_obs


OUTPUT:
output = {UCART19Blood_obs , HostT_obs, LymphTotal_obs, HostNK_obs, IL7}

*Supplementary Code 1: Monolix code of the model. UCART19 is administered on day 14 for all patients.*
